# Altered network properties in *C9ORF72* repeat expansion cortical neurons are due to synaptic dysfunction

**DOI:** 10.1186/s13024-021-00433-8

**Published:** 2021-03-04

**Authors:** Emma M. Perkins, Karen Burr, Poulomi Banerjee, Arpan R. Mehta, Owen Dando, Bhuvaneish T. Selvaraj, Daumante Suminaite, Jyoti Nanda, Christopher M. Henstridge, Thomas H. Gillingwater, Giles E. Hardingham, David J. A. Wyllie, Siddharthan Chandran, Matthew R. Livesey

**Affiliations:** 1 Euan MacDonald Centre for MND Research, University of Edinburgh, Edinburgh, EH16 4SB UK; 2 Centre for Clinical Brain Sciences, University of Edinburgh, Edinburgh, EH16 4SB UK; 3 Centre for Discovery Brain Sciences, University of Edinburgh, Edinburgh, EH8 9XD UK; 4 UK Dementia Research Institute at the University of Edinburgh, Edinburgh, EH16 4SB UK; 5 Simons Initiative for the Developing Brain, University of Edinburgh, Edinburgh, EH8 9XD UK; 6 Division of Systems Medicine, School of Medicine, University of Dundee, Dundee, DD1 9SY UK; 7 Centre for Brain Development and Repair, inStem, Bangalore, 560065 India; 8 Sheffield Institute for Translational Neuroscience, University of Sheffield, Sheffield, S10 2HQ UK

**Keywords:** Synaptic, Network, C9ORF72, ALS, FTD, Repeat expansion, Hyperexcitability, Cortical, Neuron, Electrophysiology

## Abstract

**Background:**

Physiological disturbances in cortical network excitability and plasticity are established and widespread in amyotrophic lateral sclerosis (ALS) and frontotemporal dementia (FTD) patients, including those harbouring the *C9ORF72* repeat expansion (*C9ORF72*^*RE*^) mutation – the most common genetic impairment causal to ALS and FTD. Noting that perturbations in cortical function are evidenced pre-symptomatically, and that the cortex is associated with widespread pathology, cortical dysfunction is thought to be an early driver of neurodegenerative disease progression. However, our understanding of how altered network function manifests at the cellular and molecular level is not clear.

**Methods:**

To address this we have generated cortical neurons from patient-derived iPSCs harbouring *C9ORF72*^RE^ mutations, as well as from their isogenic expansion-corrected controls. We have established a model of network activity in these neurons using multi-electrode array electrophysiology. We have then mechanistically examined the physiological processes underpinning network dysfunction using a combination of patch-clamp electrophysiology, immunocytochemistry, pharmacology and transcriptomic profiling.

**Results:**

We find that *C9ORF72*^RE^ causes elevated network burst activity, associated with enhanced synaptic input, yet lower burst duration, attributable to impaired pre-synaptic vesicle dynamics. We also show that the *C9ORF72*^*RE*^ is associated with impaired synaptic plasticity. Moreover, RNA-seq analysis revealed dysregulated molecular pathways impacting on synaptic function. All molecular, cellular and network deficits are rescued by CRISPR/Cas9 correction of *C9ORF72*^RE^. Our study provides a mechanistic view of the early dysregulated processes that underpin cortical network dysfunction in ALS-FTD.

**Conclusion:**

These findings suggest synaptic pathophysiology is widespread in ALS-FTD and has an early and fundamental role in driving altered network function that is thought to contribute to neurodegenerative processes in these patients. The overall importance is the identification of previously unidentified defects in pre and postsynaptic compartments affecting synaptic plasticity, synaptic vesicle stores, and network propagation, which directly impact upon cortical function.

**Supplementary Information:**

The online version contains supplementary material available at 10.1186/s13024-021-00433-8.

## Background

*C9ORF72* hexanucleotide repeat expansion (*C9ORF72*^*RE*^) is the most common mutation found within the ALS-FTD spectrum, giving rise to incurable, rapidly progressive and fatal disease pathologically characterised by degeneration of cortical neurons and upper and spinal motor neurons. Cortical circuit dysfunction is a consistent and prominent finding in *C9ORF72*^RE^ patients [5, 42, 50, 62]. Altered cortical network excitability is considered to be an early pathogenic driver of ALS and FTD contributing directly to excitotoxicity-mediated neurodegeneration of upper motor neurons and cortical neurons [21, 22, 36, 37, 58–60, 62]. Furthermore, clinical neurophysiological studies of *C9ORF72*^RE^ patients have demonstrated notable impairments in cortical network plasticity at the pre-symptomatic stage [5]. For many progressive neurodegenerative diseases, including ALS-FTD, functional impairments in plasticity are thought to manifest early in disease progression, being representative of altered synaptic homeostasis that precede and potentially cause neuronal dysfunction and/or loss, and lead to cognitive impairments [38, 55, 56].

Our current mechanistic understanding of potential sources of altered cortical network excitability in ALS-FTD is derived largely from mutant murine models (SOD1 and TDP-43 mutations) of ALS and ALS-FTD [17, 30, 45, 48, 65], but does not extend to provide a physiological basis for altered network excitability. Similarly, our understanding of the potential synaptic plasticity dysregulation that may occur in ALS-FTD has come from studies that use ex vivo brain slice preparations from rodent models of rare genetic mutations [24, 54]. Functional impairments in synaptic plasticity in ALS-FTD have yet to be examined in a human-based model system. Identified physiological disturbances in ALS motor neurons [55] also may provide insights into cortical neuron pathophysiology though this must remain highly tentative given that diverging potential pathophysiological mechanisms in *C9ORF72*^*RE*^ cortical and spinal neurons are established [52]. Despite its proposed pathogenicity and early prominence, cortical dysfunction in ALS-FTD remains poorly defined at both the synaptic and network level.

To address this, we have used human induced pluripotent stem cell (iPSC) derived cortical neurons from patients harbouring *C9ORF72*^*RE*^ mutations, combined with gene-edited isogenic paired lines [52], to interrogate the consequence of *C9ORF72*^*RE*^ on cortical neuronal physiology. In view of dysregulation of glutamate homeostasis being a major hypothesis underlying ALS-FTD [9, 55], we have examined physiological perturbations in iPSC-derived glutamatergic cortical neurons. We determine that *C9ORF72*^*RE*^ neurons display altered network properties that are underpinned by synaptic dysfunction, but not altered intrinsic cellular excitability, and display impairments in synaptic plasticity. Our transcriptomic analysis highlights dysregulated molecular pathways in accordance with physiological observations. Our observations are notably different from those previously reported for *C9ORF72*^*RE*^ motor neurons and provide evidence of cortical-specific pathophysiology that may contribute to cortical dysfunction in ALS-FTD.

## Methods

### iPSCs

Dermal fibroblasts from patient and control individuals were obtained under full Ethical/Institutional Review Board approval at the University of Edinburgh. Fibroblasts were reprogrammed to iPSCs by either Sendai virus or retrovirus expressing OCT4, SOX2, C-MYC, and KLF4. iPSCs were maintained in Matrigel (BD Biosciences)-coated plastic dishes in E8 medium (Life Technologies) at 37 °C and 5% CO_2_. Lines were derived from three patients harbouring repeat expansions in the *C9ORF72 *gene [52] and a healthy individual with no known association with neurodegenerative disease.

### Anterior precursor (aNPC) derivation

Human iPSCs were maintained on Matrigel (Corning), with Advanced DMEM/F12, 20% Knockout Serum Replacement, FGF-2 (10 ng/mL), L-glutamine (1 mM), 2-mercaptoethanol (100 mM) and 1% penicillin/streptomycin (P/S). All media were obtained from Life Technologies. Human iPSCs were neurally converted in suspension in chemically defined medium as described [6]. The media was changed to base media (Advanced-DMEM/F12, 1% P/S, 1% Glutamax, 1% N-2), 0.5% B-27, FGF-2 (2.5 ng/mL) upon observation of radially organised structures in neurospheres (10–21 days) and plated on Laminin (Sigma) coated tissue culture plates (Nunc) a week later. Neural rosettes were mechanically isolated, dissociated with Accutase (Sigma) and 20-40 k cells were plated in one Laminin-coated well of a 6-well plate in proliferation media (Base media, 0.2% B-27, 10 ng/mL FGF-2). aNPCs were grown to high density before passaging 1:2 with Accutase on laminin coated plates until passage 5–6 and maintained on 1:100 Reduced-growth factor Matrigel-coated plates thereafter or cryopreserved as described [6].

### Differentiation of aNPCs into cortical neuronal cultures

aNPCs were plated in default media on poly-ornithine (Sigma), laminin (Sigma), fibronectin (Sigma) and Matrigel-coated coverslips in which primary mouse astrocytes have been propagated. Primary mouse astrocytes were prepared as previously described [26]. Density of astrocytes was 100,000 per 13 mm coverslip at least 48 h prior to plating aNPCs. Cultures were fed twice a week. Default medium was supplemented with forskolin (10 μM, Tocris) from days 7–21 after aNPC platedown (200,000 per coverslip) and with BDNF and GDNF (both 5 ng/mL) from day 28 onwards. Coverslips were then processed fixed and stained as previously described [6]. Multi-electrode arrays were first coated with poly-D-lysine then the laminin, fibronectin and Matrigel-coating was applied to the region containing the electrode arrays (60MEA200/30iR-Ti, Multi Channel Systems). aNPCs were plated to the coating spot and left for 2 h to adhere. Array wells were then flooded with default media containing suspended DIV14 mouse astrocytes.

### Immunohistochemistry

Five–six weeks old cultures on glass coverslips were fixed in 4% PFA at room temperature (RT) for 20 min. They were permeabilised with 0.1% tritonX-100, blocked with 6% goat serum and stained with primary antibodies against βIII-tubulin (dilution 1:500, Sigma), human nuclei (dilution 1:200, Millipore), nestin (dilution 1:200, Millipore), GFAP (1:400, Sigma), synapsin-1 (dilution 1:500, Sigma) and PSD-95 (dilution 1:250; Neuromab) sequentially for 2 h at RT. These were then probed with appropriate secondary antibodies and mounted with FluorSave and imaged in Zeiss LSM Z10 confocal microscope using 63X objective. For synaptic density analysis, 5 fields of 20 μm region across 3 coverslips were analysed for the co-localised puncta of synapsin-1 and PSD-95 using colocalization plugin in ImageJ.

### RNA extraction, RNA sequencing and transcriptomic analysis

Total RNA was extracted from cortical neurons from 2 independent isogenic corrected paired cell lines at day 35 post platedown using RNeasy Mini kit (Qiagen), according to the manufacturer’s instructions. RNA samples were assessed for concentration (NanoDrop ND-100 Spectrometer, NanoDrop Technologies) and quality (Agilent 2200 Tapestation, Agilent Technologies) before library preparation. Library preparation and sequencing were carried out by Edinburgh Genomics (Edinburgh, UK). For each sample, cDNA was converted to a sequencing library using the TruSeq stranded mRNA-seq library. Barcoded libraries were pooled and sequenced on an Illumina HiSeq 4000 using 75 base paired-end reads to generate at least 111 million raw reads per sample. The reads were mapped to the primary assembly of the human (hg38) reference genome contained in Ensembl release 90 [12]. Alignment was performed with STAR, version 2.5.3a [16]. Tables of per-gene read counts were generated from the mapped reads with featureCounts version 1.5.2 [33]. Differential gene expression analysis, using DESeq2 version 1.18.1, specifically examined the intersection in commonly and concordantly differentially expressed genes between the two mutant-isogene pairs, using a false discovery rate of 20%, achieved by setting a Benjamini-Hochberg corrected *p*-value threshold of 0.2 (genes with an average FPKM < 1 were disregarded). Gene ontology (GO) analysis was performed on all the differentially expressed genes to identify putatively altered pathways or processes using topGO version 2.30.1 [1]. RNA-seq data are available upon request to the corresponding authors.

### Morphology

Cortical NPCs were sparsely transduced with lentivirus expressing GFP in order to label individual cells for analysis (ca. 1 viral particle to 10 cells). Following labelling with GFP, NPCs were differentiated as mentioned above and immunohistochemistry was performed against GFP and β3-tubulin. These were then probed with appropriate secondary antibodies and mounted with FluorSave and imaged in Zeiss LSM Z10 confocal microscope using 20X objective. Total neurite length (sum of all the processes) in the GFP channel was manually traced using ImageJ.

### Multi-electrode array (MEA) electrophysiology

Extracellular recordings from 59 channels per array were acquired at 37 °C in the culture media using a Multi Channel Systems MEA system at a sampling rate of 20 kHz. Data was analysed using the Multichannel Systems software and in-house custom Matlab scripts.

### Patch-clamp electrophysiology

For other electrophysiological experiments, whole-cell patch-clamp recordings were performed as described [6, 34] using electrodes filled with (in mM): 155 K-gluconate, 2 MgCl_2_, 10 Na-HEPES, 10 Na-PiCreatine, 2 Mg_2_-ATP, and 0.3 Na_3_-GTP, pH 7.3, 300 mOsm. For spontaneous action potential activity, cells were typically bathed in an extracellular recording comprising (in mM): 152 NaCl, 2.8 KCl, 10 HEPES, 2 CaCl_2_, 10 glucose, pH 7.3, 320–330 mOsm. For mEPSC recordings, the extracellular solution was supplemented with TTX (1 nM), picrotoxin (50 μM) and MgCl_2_ (1.5 mM). For intrinsic membrane and excitability properties, the extracellular solution was supplemented with CNQX (5 μM) and D-APV (50 μM). Recordings were performed at room temperature (20-23 °C). Current and voltage measurements were typically low-pass filtered online at 2 kHz, digitized at 10 kHz and recorded to computer using the WinEDR V2 7.6 Electrophysiology Data Recorder (J. Dempster, Department of Physiology and Pharmacology, University of Strathclyde, UK; www.strath.ac.uk/Departments/PhysPharm/). Series resistance compensation was applied up to 75%. Recordings were omitted from analysis if the series resistance changed by more than 20% during the experiment, or if they exceeded 20 MΩ.

### Burst analysis

Burst detection for both single cells and MEAs were performed using custom-written Matlab scripts. For patch-clamp recordings action potentials were identified using threshold detection (routinely set at − 10 mV) and bursts were defined as groups of action potentials with a minimum inter burst period set as log10 of the intra spike interval. For each MEA, 4–10 active channels were selected for further analysis. Bursts were identified as activity 2–5 times the standard deviation of the baseline (as determined by the signal-to-noise ratio) with a minimum quiet period set to define separate bursts. This was routinely set to 5 s given the lowest observed inter burst period was 9.6 s. The spike threshold was variable, but consistent between each of our isogenic and C9 pairs for each experiment and optimal to detect as many, but variable, number of channels with robust activity. On all MEAs there  were both active and inactive channels (likely due to some electrodes not having active cells close enough) but all the active channels showed the same pattern of activity with low standard deviation in the burst start times across channels ranging from 0.10 to 0.78 s, indicating a synchronous network across the area of the MEA electrodes.

### mEPSC analysis

mEPSC recordings were analysed offline using the WinEDR software stated above. A dead time window of 10 ms was set and individual mEPSCs were detected using an algorithm that selected for mEPSCs below a − 4 to – 6 pA amplitude threshold and greater than 1 ms in duration. mEPSCs that had a monotonic rising phase with a 10–90 rise time of lower than 6 ms and a Ƭ-decay with a decay time constant of lower than 25 ms were selected for analysis. Recordings were then visually inspected for validity. For mEPSC analysis (Fig. 2c), data were obtained from at least 2-min recordings and neurons that displayed mEPSC frequencies under 0.05 Hz were omitted from the analysis. For sucrose experiments, baseline mEPSC properties were determined from a 2-min stretch of mEPSC activity of at least 0.05 Hz. The transient phase was determined from the onset of sucrose application to the transition of mEPSC activity to steady-state activity. Steady-state data were determined from at least a 30 s stretch of mEPSC activity in continued presence of sucrose.

### Statistical analysis

Statistical analysis was performed using GraphPad Prism software. Data are represented as mean ± s.e.m. *p < 0.05, **p < 0.01, ***p < 0.001. The number of experimental replicates (for MEA recordings, this indicates number of plates; for patch-clamp recordings this indicates number of cells) is denoted as n and N represents the number of independent de novo preparations of batches from which n is obtained. Data were initially determined to be parametric or non-parametric before applying either one-way ANOVA with Bonferroni’s multiple comparisons test or unpaired t-tests or Welch’s t-test, as appropriate.

## Results

### C9ORF72^RE^-derived cortical neurons display network dysfunction

iPSCs from three patients harbouring *C9ORF72*^RE^ mutations (*C9–1,2,3*), three paired isogenic control lines (*C9-Δ1,2,3*) in which the *C9ORF72*^RE^ mutation had been selectively excised by CRISPR/Cas9-mediated gene-editing [52], and an unrelated healthy individual (*Con*) were used to generate cultures of excitatory cortical neurons using an established protocol [6, 52]. Cortical neurons were maintained in co-culture with primary mouse astrocytes in order to promote neuronal maturation [35] and experiments were performed at 4-to-6 weeks post differentiation. Cultures efficiently differentiated into enriched populations of neurons by this time point (Supplementary Figure 1) and presented a marker profile consistent with a glutamatergic cortical neuron identity (Supplementary Figure 2).

**Fig. 1:**
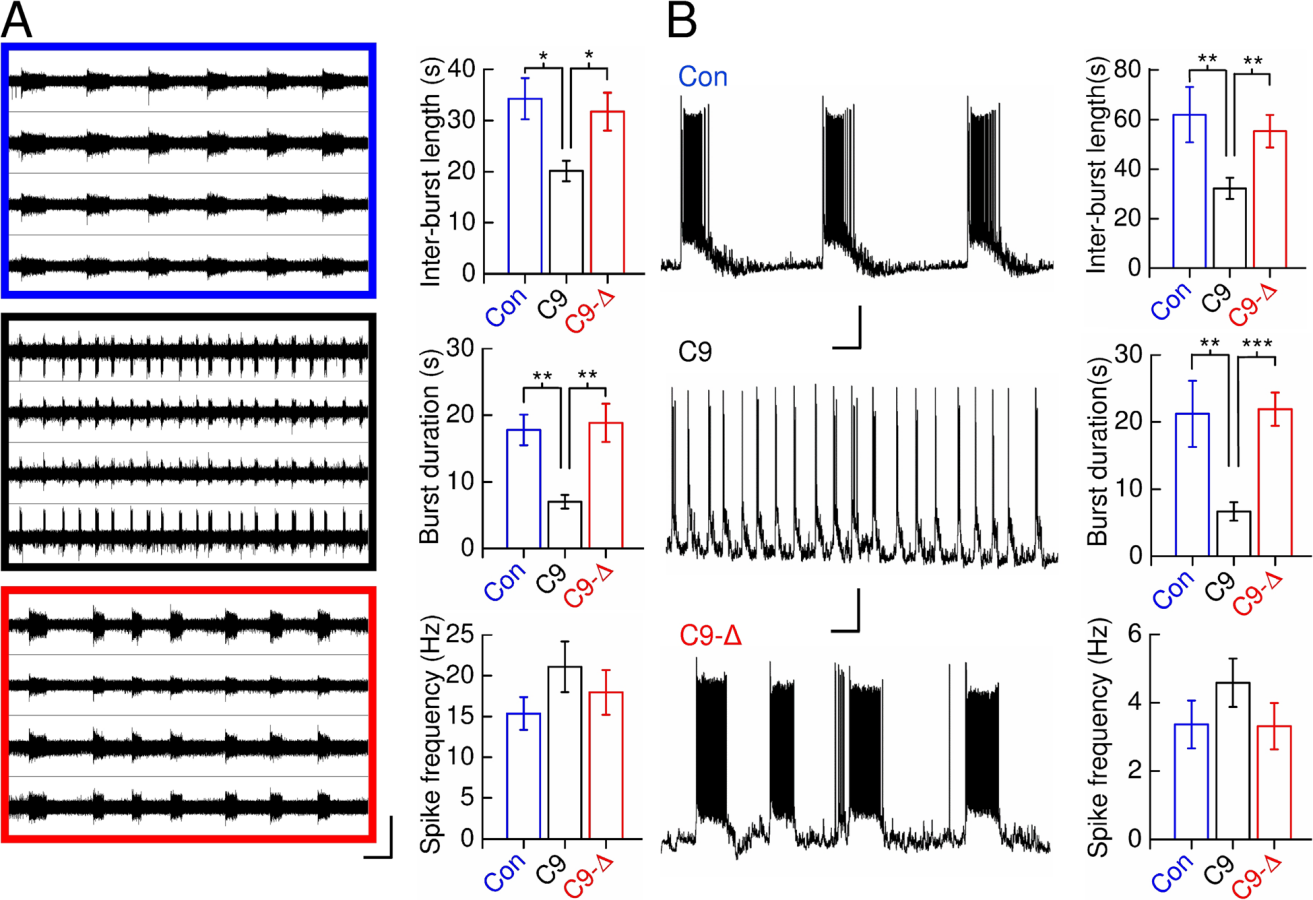
Altered network function in *C9ORF72*^RE^-derived cortical neurons. **a** *Left*, Representative MEA recordings from control (*blue*), *C9ORF72*^RE^- (*black*) and respective *C9ORF72*^RE^ gene-edited- (*red*) derived neuronal cultures each showing traces from four electrodes from single arrays. Note the difference in the network burst properties in *C9ORF72*^RE^-derived cultures. Scale bar, 50 μV, 30 s. *Right*, Mean (± s.e.m.) data for control (*Con*), *C9ORF72*^RE^- (*C9*) and respective *C9ORF72*^RE^ gene-edited- (*C9-Δ*) derived neurons for interburst and network burst lengths (Con, *N* = 9; C9, *N* = 10; C9-Δ, *N* = 9) and intra-network burst spiking frequencies (Con, *N* = 7; C9, *N* = 6; C9-Δ, *N* = 8). Data are pooled for C9 and C9-Δ – individual pair comparisons are presented in Supplementary Figure 4A. **b** *Left*, Representative current-clamp recordings from control, *C9ORF72*^RE^- and respective *C9ORF72*^RE^ gene-edited-derived neurons (cells were held at − 74 mV; scale bar, 20 mV, 30 s). *Right*, Mean (± s.e.m.) data for control (*Con*), *C9ORF72*^RE^- (*C9*) and respective *C9ORF72*^RE^ gene-edited- (*C9-Δ*) derived neurons for interburst lengths, burst lengths and intraburst spiking frequencies (Con, *n* = 10, *N* = 4; C9, *n* = 16, *N* = 4; C9-Δ, *n* = 13, *N* = 4). Data are pooled for C9 and C9-Δ – individual pair comparisons are presented in Supplementary Figure 4B. Note that differences in burst properties between MEA and patch-clamp experiments are likely to reflect differing resting membrane potentials and recording temperatures at which experiments are conducted and/or the alternate plating order of neurons and astrocytes for each respective approach. Significance determined by one-way ANOVA with Bonferroni’s post hoc test

To examine network dysfunction in *C9ORF72*^RE^ excitatory cortical neurons we initially used a multi-electrode array (MEA) recording platform. *C9ORF72*^RE^ excitatory cortical neurons displayed an increase in the rate of burst firing (reduced network burst duration and inter-burst lengths versus healthy and isogenic controls) (Fig. 1a). Although shorter inter-burst lengths are typically associated with increased glutamate-mediated excitatory network activity, the reduced network burst duration in *C9ORF72*^RE^ cortical neurons (Fig. 1a) is inconsistent with this [3, 43]. Intra-burst spike frequency was comparable across all lines (Fig. 1a) suggesting that intrinsic excitability is unaffected. We then examined whether spontaneous network activity was maintained at the single-cell level using whole-cell patch-clamp recording. Burst activity from *C9ORF72*^RE^ neurons also showed a similarly reduced inter-burst length and burst duration and no change in intra-burst spike frequency versus controls (Fig. 1b). Spontaneous burst firing was blocked by CNQX and was not affected by bicuculline consistent with an enriched population of excitatory glutamatergic neurons and the absence of GABA-ergic interneurons (Supplementary Figure 3 [6, 34, 52];). Our data show that network activity is altered in *C9ORF72*^RE^ cortical neurons.

A possible determinant of altered network excitability may be due to differences in the intrinsic membrane properties and intrinsic capacity of *C9ORF72*^RE^ cortical neurons to fire and maintain action potentials. Previously, our work and others has shown such properties to be altered in *C9ORF72*^RE^ motor neurons [14, 49, 61, 66], but remains to be described in the context of *C9ORF72*^RE^ cortical neurons. Our network analysis suggests no difference in intrinsic excitability, so we therefore directly examined these intrinsic properties by performing whole-cell current-clamp recordings in the presence of blockers of fast synaptic transmission. Our measurements of intrinsic membrane properties (resting membrane potential, input resistance and whole-cell capacitance) and the generation of action potentials in response to depolarisation are comparable across all lines (Supplementary Figure 5). These data are collectively consistent with intrinsic membrane and excitability properties not being a determinant of altered network activity in *C9ORF72*^RE^ neurons.

### C9ORF72^RE^-derived cortical neurons have increased synaptic input

**Fig. 2:**
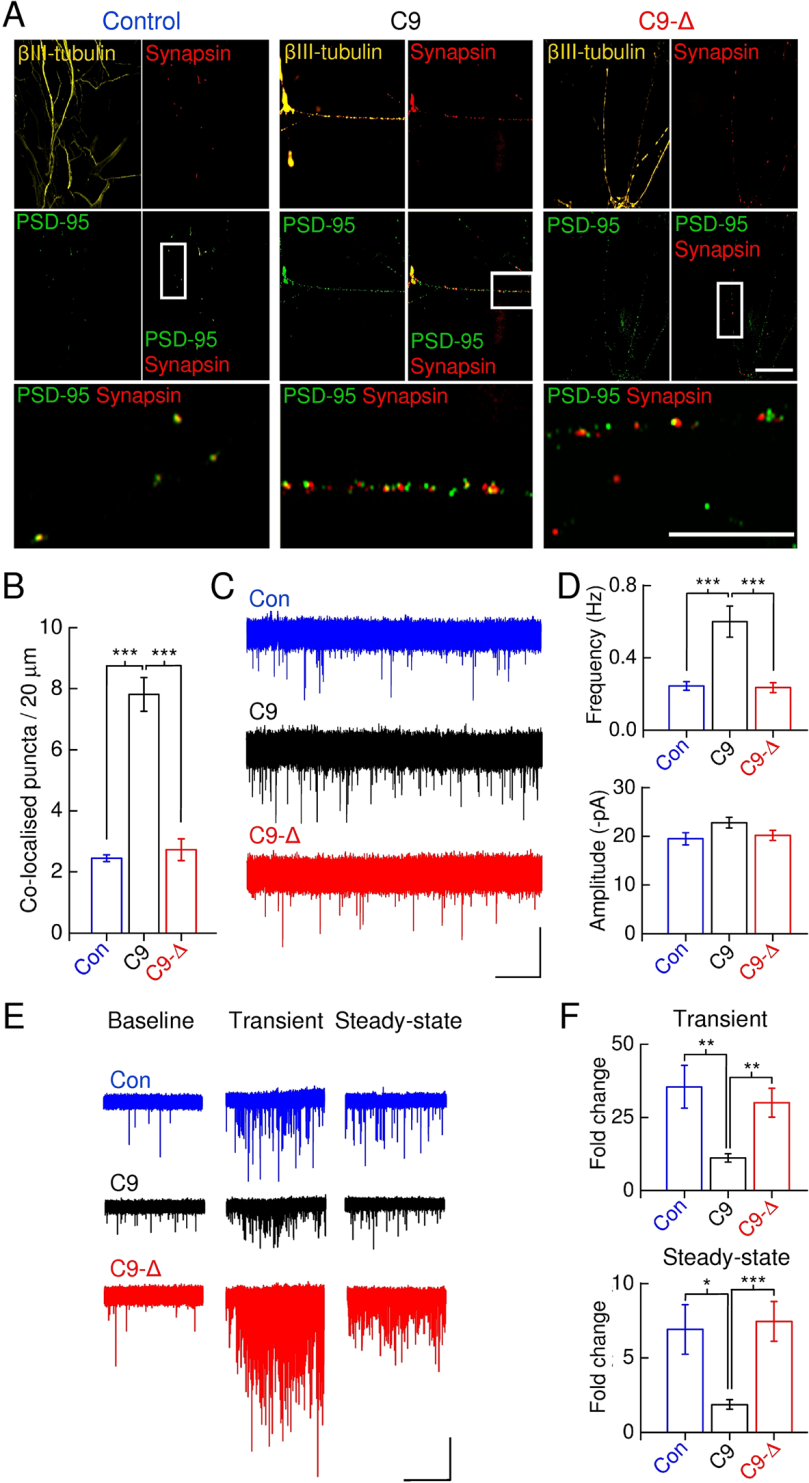
Synaptic density, mEPSC properties and pre-synaptic dysfunction. **a** For each line, [control, *C9ORF72*^RE^- (C9) and *C9ORF72*^RE^ gene-edited- (C9-Δ) cortical neurons], low magnification images (scale bar, 20 μm) show immunostaining for βIII-tubulin (*yellow, top left*), synapsin-1 (*red*), PSD-95 (*green*) and a PSD-95/synapsin-1 composite view (*lower*) from which the region of interest is highlighted. The region of interest is shown with greater higher magnification image below (scale bar, 10 μm). **b** Mean ± s.e.m. co-localised PSD-95/synapsin-1 puncta per 20 μm of neurite length for each line type (Control, *N* = 3; C9, *N* = 12; C9-Δ, *N* = 12). Data for C9 and C9-Δ are pooled from three respective *C9ORF72*^RE^- and gene-edited pairs – individual pair comparisons are presented in Supplementary Figure 6A. Data from each de novo preparation represents a mean obtained from four coverlips each consisting of five randomly selected image fields. **c** Sample traces from recordings of mEPSC events from control, *C9ORF72*^RE^- and *C9ORF72*^RE^ gene-edited-cortical neurons. Recordings were made at a holding potential of − 84 mV. Scale bar; 20 pA, 10 s. mEPSCs in all lines are mediated by Ca^2+^-impermeable (NASPM-insensitive) AMPA receptors (CNQX-sensitive). **d** Mean ± s.e.m. mEPSC frequency and amplitude for each line (Control, *n* = 14, *N* = 3; C9, *n* = 52, *N* = 12; C9-Δ, *n* = 42, *N* = 11). Data are pooled for C9 and C9-Δ – individual pair comparisons are presented in Supplementary Figure 7. **e** Sample traces from recordings of mEPSC events before and in the presence of sucrose from control (Con), *C9ORF72*^RE^-(C9) and *C9ORF72*^RE^ gene-edited (C9-Δ)-cortical neurons. The transient and steady-state phases evoked by sucrose addition are highlighted. Note the difference in mEPSC frequency in these phases versus baseline. Scale bar, 50 pA, 5 s. **f** Mean ± s.e.m. fold change in mEPSC frequency for each line for the transient (Control, *n* = 14, *N* = 3; C9, *n* = 28, *N* = 10; C9-Δ, *n* = 30, *N* = 9) and steady-state phases (Control, *n* = 8, *N* = 2; C9, *n* = 23, *N* = 8; C9-Δ, *n* = 23, *N* = 8). Data are pooled for C9 and C9-Δ – individual pair comparisons are presented in Supplementary Figure 8. Significance of data in the figure determined by one-way ANOVA with Bonferroni’s post hoc test

To determine whether the altered network activity in *C9ORF72*^RE^-derived cortical neurons was of synaptic origin we undertook quantification of the co-localisation of pre- (synapsin-1) and post-synaptic (PSD-95) markers and observed an increase in synaptic densities on *C9ORF72*^RE^ versus control neurons (Fig. 2a, Supplementary Figure 6A). Increased synaptic density may reflect an increase in neuronal morphology and therefore we examined the neurite length in our cultures (Supplementary Figure 6B, C). However, consistent with our whole-cell capacitance measurements, our data did not reveal any morphological changes that could account for the large change in synaptic density in *C9ORF72*^*RE*^ neurons. Consistent with these data we also found an increase in the frequency of mini excitatory postsynaptic currents (mEPSCs) in *C9ORF72*^RE^ neurons compared to isogenic controls and healthy volunteer-derived neurons (Fig. 2c, d, Supplementary Figure 7). AMPAR-mediated mEPSC amplitudes and kinetics (rise-times and decay-times), and further the expression of AMPA receptor subunits, were not changed indicating that the properties and expression of synaptic AMPA receptors were not altered (Fig. 2c, d, Supplementary Figure **7**). These findings indicate that *C9ORF72*^RE^ cortical neurons display increased synaptic density resulting in elevated synaptic input.

### C9ORF72^RE^–derived cortical neurons exhibit pre-synaptic dysfunction

Increased pre-synaptic glutamate release could also underlie an increased mEPSC frequency and a central determinant of release properties and network burst properties is the size of the vesicle readily releasable pool (RRP) [10, 32]. The RRP size was functionally estimated using hypertonic sucrose, an established method to generate Ca^2+^-independent exocytosis of the vesicular RRP [40, 47]. We observed sucrose-application-evoked mEPSC activity that can be classified into an initial transient phase (initial depletion of the RRP) and a steady-state phase (on-going replenishment of the RRP; Fig. 2e and Supplementary Figure 8A [40];). Given that conventional measurements of RRP are determined by the integral of the total evoked current and are directly proportional to synaptic density we have examined the fold change in mEPSC frequencies because *C9ORF72*^RE^ cortical neurons exhibit increased synaptogenesis over control lines. A fold reduction in mEPSC frequency (Fig. 2f, Supplementary Figure 8B), but not their amplitude (Supplementary Figure 8C), was observed in *C9ORF72*^RE^ neurons compared to control lines for both the transient and steady-state phases. Thus, despite an observed increase in mEPSC frequency during baseline recordings, *C9ORF72*^RE^ cortical neurons display a functionally reduced RRP size that is replenished at a slower rate. These data potentially explain the shorter network burst durations in *C9ORF72*^RE^ cortical neurons.

Physiological vesicular release is Ca^2+^-dependent and thus we examined this by measuring fold changes in mESPC frequencies before and after addition of KCl. No differences in the fold change of mEPSC frequencies were found between *C9ORF72*^RE^ versus paired isogenic control neurons (Supplementary Figure 9). These data suggest that overall depolarisation-mediated Ca^2+^-evoked release is equivalent in the pre-synaptic terminals of each line. Nonetheless our data indicate that the RRP size is impacted in *C9ORF72*^RE^ cortical neurons.

### Synaptic potentiation in C9ORF72^RE^ cortical neurons is impaired

**Fig. 3:**
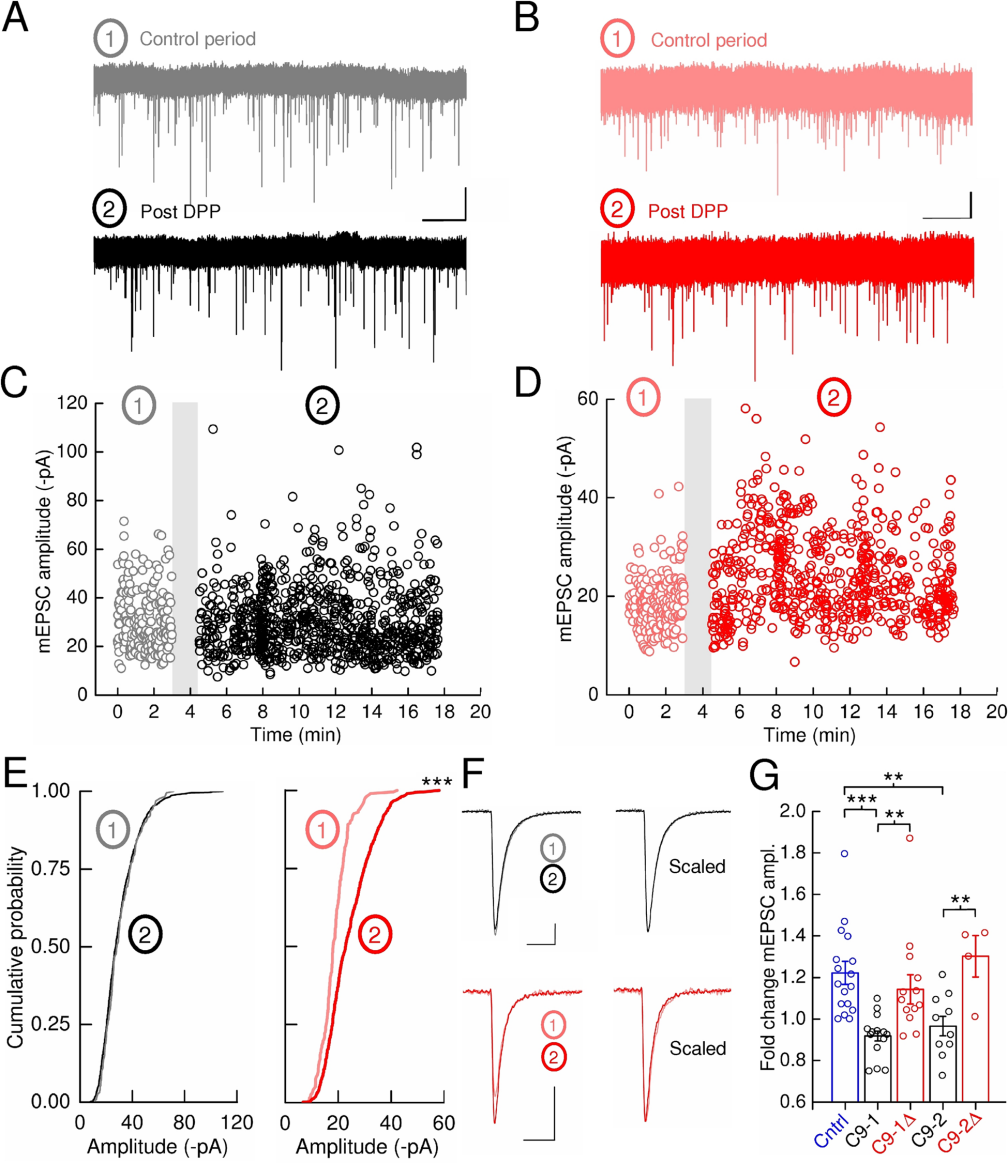
Synaptic plasticity. **a** and **b** Example recordings of mEPSCs prior to and after DPP (10 depolarising pulses of 3 s in duration, every 9 s, from − 84 to + 16 mV) for *C9ORF72*^RE^ (1. *grey* and 2. *black*, respectively; scale bar; 20 pA, 5 s) and *C9ORF72*^RE^-Δ (1. *light red* and 2. *red*, respectively; scale bar; 10 pA, 5 s) neurons. **c**, **d** Individual mEPSC amplitude plots for DPP experiments performed upon *C9ORF72*^RE^ and *C9ORF72*^RE^-Δ neurons, respectively. For *C9ORF72*^RE^ neurons, mEPSCs before (1) and after DPP (2) are represented in *grey* and *black*, respectively. For *C9ORF72*^RE^-Δ neurons, mEPSCs before (1) and after DPP (2) are represented in *light red* and *red*, respectively. Note the lack of potentiation in *C9ORF72*^RE^, but not *C9ORF72*^RE^-Δ, neurons. **e** Cumulative probability plots of mEPSC amplitudes for the initial control period (1) and after DPP (2) of the data shown in C and D. Significance of shift of mEPSC data (Kolmogorov–Smirnov test); *C9ORF72*^RE^, *p* = 0.325; *C9ORF72*^RE^-Δ, *p* < 0.001. **f** *Left*, mean mEPSCs for data shown in B (*C9ORF72*^RE^) and C (*C9ORF72*^RE^-Δ) for initial control period (1) and post DPP (2). Scale bars; 5 pA, 5 ms. *Right*, mean scaled mEPSCs. **g** Mean ± s.e.m. fold increase of mEPSC amplitude 10 minutes post-DPP from initial control period for control (*n* = 17), C9–1 (*n* = 14), C9–1Δ (*n* = 13), C9–2 (*n* = 4) and C9–2Δ (*n* = 10). Example traces and data for the control line are presented in Figure S10. Significance determined by unpaired t-tests (*C9ORF72*^RE^ versus *C9ORF72*^RE^-Δ) and one-way ANOVA with Bonferroni’s post hoc test (control versus *C9ORF72*^RE ^)

Cortical network plasticity in *C9ORF72*^RE^ patients is impaired and suggests that this is due to perturbed activity-dependent synaptic plasticity [5]. The activity-dependent potentiation of AMPA receptor-mediated mEPSCs is a central feature of classical models of synaptic plasticity and therefore we applied a series of depolarizing voltage steps to our neurons, as previously described, that leads to a potentiation of the amplitude of mEPSC in rodent hippocampal neurons [4, 29, 31, 63, 64]. Following this depolarisation pulse protocol (DPP), we observed that mEPSCs in control neurons were transiently potentiated by around 20% in amplitude from the initial control period before returning close to control levels (Supplementary Figure 10). No significant shift in mEPSC amplitude was observed when no stimulation was applied. Consistent with previous studies on primary hippocampal neurons [4], we found this mEPSC potentiation to be dependent upon the activation of voltage-gated Ca^2+^ channels and intracellular elevations in Ca^2+^ as was blocked by voltage-gated Ca^2+^ channel antagonist, nifedipine, and supplementation of Ca^2+^ chelator BAPTA to the patch pipette, respectively (Supplementary Figure 10E, Supplementary Figure 11A-F). In contrast, we observed that DPP did not induce any potentiation of mEPSC amplitudes in *C9–1* and *C9–2* neurons (Fig. 3a, c, e-g). The respective isogenic *C9–1Δ* and *C9–2Δ* neurons displayed a significant mEPSC potentiation post-DPP (Fig. 3b, d-g). The mEPSC decay time constant did not change post-DPP with respect to baseline activity in either control, C9 or C9-*Δ* neurons suggesting that the composition of AMPA receptors mediating mEPSCs is unchanged post-DPP (Fig. 3f; Supplementary Figure 11G). These data are therefore consistent with a *C9ORF72*^RE^-mediated physiological disruption of mEPSC potentiation in *C9ORF72*^RE^-derived cortical neurons in our model of synaptic potentiation.

### RNA sequencing highlights molecular disruption at the synapse in C9ORF72^RE^ cortical neurons

**Fig. 4:**
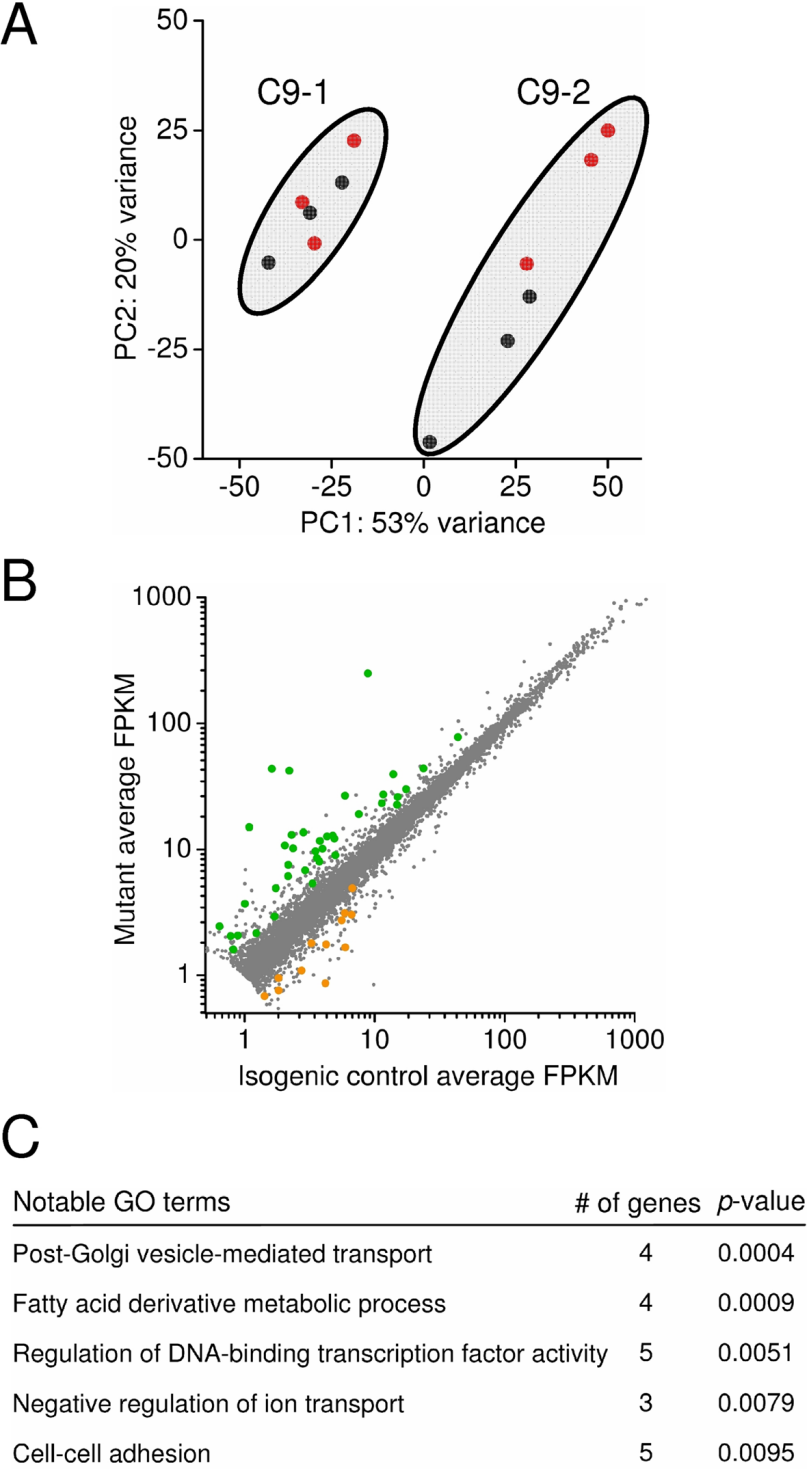
Transcriptomic analysis of *C9ORF72*^RE^ cortical neurons. **a** Principal component analysis of gene expression derived from RNA sequencing from *C9ORF72*^RE^ cortical neurons (C9–1, C9–2; black) and isogenic gene-corrected control cortical neurons (C9–1Δ, C9–2Δ; red). Each data point represents a de novo differentiation of cortical neurons. As highlighted, the isogenic controls cluster accordingly with their respective parental *C9ORF72*^RE^ lines. **b** Scatter plot showing comparison of gene expression (as average Fragments Per Kilobase of transcript per Million mapped reads) from cortical neurons derived from two independent *C9ORF72*^RE^ iPSC lines and their corresponding isogenic controls. Green and orange data points denote the overlap of significantly up- and down-regulated genes in both mutant-correction pairs, respectively (false discovery rate, *p* < 0.2). **c** Selected gene ontology terms enriched in dysregulated genes

To begin to understand the molecular changes that underpin the observed physiological dysfunction in *C9ORF72*^RE^ cortical neurons, we next performed transcriptomic analysis of cortical neurons derived from two independent *C9ORF72*^RE^ iPSCs (C9–1 & C9–2) and their corresponding isogenic controls (C9-Δ1 & C9-Δ2). Principal component analysis (PCA) of gene expression showed segregation of differential gene expression *between* the two mutant-isogenic pairs, though a high degree of similarity *within* a mutant-isogene pair, as expected (Fig. 4a). We therefore assessed our data set in order to determine common dysregulated gene expression between the different lines employed (Fig. 4b, Supplementary Table 1). Our biological process gene ontology analyses (Fig. 4c) revealed dysregulated expression of genes involved in vesicle regulation (*Gopc*, *Vamp5*), cell-cell adhesion (*Cbln1*, *Pcdhgc4*), negative regulation of ion transport (*Htr2a*) fatty acid metabolism and regulation of DNA-binding transcription factor activity (*Irak1*, *Sigirr*). These novel transcriptomic data reveal dysregulated multiple pathways in *C9ORF72*^RE^ cortical neurons that may contribute to the observed synaptic dysfunction.

## Discussion

Increased synaptic glutamate transmission within the cortex presents an attractive hypothesis to potentially explain cortical network hyperexcitability present in early symptomatic *C9ORF72*^RE^ patients [42, 50, 62] and ALS in general [22]. Our data provide a human in vitro mechanistic exploration of physiological impairments in *C9ORF72*^RE^ patient-derived excitatory cortical neurons that reveal that perturbed network activity is underpinned by functional synaptic alterations that impact upon excitability. Furthermore, noting that ALS-FTD patients exhibit impairments in network plasticity, we have determined that *C9ORF72*^*RE*^ cortical neurons exhibit impairments in synaptic plasticity. Importantly, the physiological alterations observed in iPSC-derived *C9ORF72*^RE^ cortical neurons are disparate from that previously observed in iPSC-derived *C9ORF72*^RE^ motor neurons where intrinsic excitability appears to be primarily affected [55]. Our data reveal that intrinsic excitability is not affected in *C9ORF72*^RE^ cortical neurons.

An increase in network burst frequency in *C9ORF72*^RE^ patient-derived excitatory cortical neurons is highly consistent with a mechanism requiring increased excitatory input. Our data demonstrate that *C9ORF72*^RE^ cortical neurons display an increased synaptic input as a result of an increased synaptic density. Such findings are broadly consistent with murine models of ALS, where increased synaptic input of excitatory cortical neurons are observed in pre-symptomatic mutant TDP-43 mice ([17]; but see [25]) and SOD^G93A^ mice [17, 48, 57]. Cortical neurophysiological impairments were not found in a *C9ORF72*^RE^ murine model though this model does not display classical ALS-FTD pathology or neurodegeneration [44]. Our transcriptomic approach has revealed potential causes to this increase in synaptic density. PCDHGC4, a γ-protocadherin, negatively regulates the function of neuroligin-1, a post-synaptic molecule that interacts with pre-synaptic neurexin to maintain and promote synapse structures in forebrain neurons [39]. Reduced expression of PCDHGC4 in our *C9ORF72*^RE^ cortical neurons is therefore compatible with increased neuroligin-1 function and increased synaptic density. Overexpression of neuroligin-1 has previously been shown to increase excitatory synaptic activity in in vitro cortical neurons [8]. Equivalently, CBLN1, is a pre-synaptically expressed molecule that interacts with neurexins and promotes synaptogenesis [51] and is upregulated in *C9ORF72*^RE^ cortical neurons. Contrastingly, CBLN1 has been reported to be downregulated in *C9ORF72*^RE^ iPSC-derived motor neurons [49]. Together, these studies indicate that increased cortical glutamate-mediated synaptic input is an early feature of ALS. Future work will require to determine when increased synaptic density alongside altered network excitability presents in ALS progression in patients.

Many ALS-focused studies describing altered glutamatergic input have examined synaptic function without assessing presynaptic function in detail, nor have they examined the consequences for network activity. An increase in excitatory synaptic input might be expected to increase network burst duration in addition to burst frequency [32]. However, our assessment of network activity revealed a decrease in network burst duration and appears to be consistent with a decrease in glutamatergic synaptic transmission. Consistent with this, our evaluation of pre-synaptic function revealed an estimated reduced size and replenishment of vesicular RRP. Importantly, a reduced RRP size and replenishment rate has been previously shown to generate early burst termination to shorten burst duration [10, 32]. This provides the most parsimonious explanation of the observed shorter network burst duration in *C9ORF72*^RE^ cortical neurons. A disruption in the vesicular RRP suggests mechanisms in which synaptic vesicular trafficking are impaired. Noting that C9ORF72 is detected in pre-synaptic terminals, our data resonate with previous studies highlighting the role of C9ORF72 protein in vesicular trafficking within the trans-Golgi network and endosomal signalling and suggest that *C9ORF72* haploinsufficiency may result in a reduced RRP [2, 19, 53]. Our transcriptomic data provide further evidence of dysregulated genes associated with impaired vesicular trans-Golgi network and endosomal signalling in ALS, consistent with a growing body of evidence of impaired vesicular trafficking in ALS that may impact on the RRP size [11, 15, 46]. For example, our data set highlights an upregulation of the *GOPC* gene, a chaperone protein that is expressed across the trans-Golgi network and endosomes. Amongst many interactions, GOPC is associated with syntaxin-6 that regulates endosomal vesicular transport [7]. Furthermore, TDP-43 protein appears to bind GOPC RNA [41]. Collectively, our data show a reduction in the RRP that is consistent with impairments in vesicular trafficking.

Importantly, vesicular release is typically stimulated via Ca^2+^-dependent mechanisms. Despite a reduced RRP, our evaluation of depolarisation/Ca^2+^-dependent vesicular release appears to be equivalent in *C9ORF72*^RE^ excitatory cortical neurons versus isogenic controls. Indeed, our afterhyperpolarisation (AHP) data suggest that calcium mediated influx is not impacted to influence intrinsic excitability properties. However, we must remain cautious that AHP and exocytosis could be independently calcium regulated processes in our cells, subject to localised intracellular calcium regulation. One potential explanation could be that localised Ca^2+^-dependent mechanisms controlling vesicular release in *C9ORF72*^RE^ excitatory cortical neurons are enhanced over control lines to generate the higher release probability required to elevate the fold increase in mEPSC frequency to comparable levels to the control lines. Dysregulated cytoplasmic Ca^2+^ levels in *C9ORF72*^RE^-derived motor neurons have been previously reported [13, 28] and this elevation in Ca^2+^ levels may contribute to an increased release probability. However, our findings contrast with those of Jensen et al. [28] who suggest that KCl-stimulated release is impaired, due to a GA-driven loss of the protein SV2, in *C9ORF72*^RE^ patient-derived cortical neurons.

Our data show that synaptic potentiation in *C9ORF72*^RE^ excitatory cortical neurons is impaired. Notably, transcranical magnetic stimulation-based studies show both presymptomatic and post-symptomatic *C9ORF72*^RE^ patients exhibit an abolishment of activity-dependent cortical network plasticity [5]. Together, these data suggest impairments in functional synaptic plasticity may emerge as an early pathophysiological event in *C9ORF72*^RE^–mediated disease progression to impair network plasticity. The pathological determinants of the impairments in synaptic plasticity remain unknown in *C9ORF72*^*RE*^ cortical neurons, though a very recent study has shown synaptic plasticity impairments in murine C9orf72 knock out animals [27], suggesting a role for the C9orf72 protein in plasticity mechanisms. In addition to our own data set indicating impact upon synaptic physiology, data sets highlight that altered gene expression in *C9ORF72*^RE^ patient tissue [46] and disrupted cell signalling pathways in iPSC-derived *C9ORF72*^RE^ neurons [13] are implicated in synaptic plasticity. Furthermore, recent transcriptomic work has associated the expression of di-peptide repeat proteins with a reduction in expression of a mediator of synaptic plasticity [15]. Our data therefore firmly determines synaptic plasticity impairments are present in human *C9ORF72*^*RE*^ cortical neurons.

Crucially, C9ORF72^RE^ mutations are causal to both ALS and FTD. In this regard, we note that the vast majority of clinical pathophysiological measurements describing hyperexcitability are made from the motor cortex, which is primarily affected in ALS [22]. Nonetheless cortical hypexcitability is evidenced in rodent models of FTD [20]. We acknowledge that our data may have preferential relevance for FTD over ALS, or vice versa. This is likely to become more apparent with increased pathophysiological characterisation of *C9ORF72*^*RE*^ ALS-FTD patients. Further, early identification of pre-symptomatic individuals and longitudinal stratification of these observations will allow us to place further confidence upon the pathophysiological staging that our observations are most likely to mirror. Our data represent one in vitro time point, but as discussed, closely align with aspects of pre-symptomatic cortical neuron impairments evidenced in rodent models of ALS and ALS-FTD. Importantly, the network excitability is not investigated in these models. Our data suggest that increases in synaptic transmission may lead to increased network burst frequency, however at the same time, indicate that other concurrent processes reduce burst activity, reflective of multiple changes in the molecular landscape of synaptic function, potentially in an attempt to homeostatically compensate against other network impairments. Indeed, work performed more extensively in the context of Alzheimer’s disease has established that homeostatic network activity adaptation, including impaired synaptic plasticity, is an early feature and precedes that of pathophysiological network failure likely resulting in the manifestation of the clinically observed network hyperexcitability [18]. In this regard, there are intriguing similarities in our data set to cortical neurons derived from Alzheimer’s patient iPSCs that display increased synaptic activity at the same time point [23].

.

## Conclusions

In summary, our study provides physiological evidence supporting the involvement of widespread glutamatergic synaptic dysfunction as a potential pathogenic mechanism of *C9ORF72*^RE^-mediated disease, a disease that has considerable impact in cortical neurons in addition to motor neurons. We reveal that pre and post-synaptic defects are highly prominent in cortical neurons in ALS-FTD and are suggested to combine to generate early network excitability alterations and impaired synaptic plasticity. We note these are very different physiological perturbations previously reported for motor neurons derived from *C9ORF72*^*RE*^ patients and therefore this study shows that the pathophysiological processes in different brain regions are likely to show divergence. These early synaptic defects are likely linked to core mechanisms of neurodegenerative disease progression and symptoms in ALS-FTD.

## Supplementary Information

**Additional file 1: Supplementary Figure 1.** Neuronal specification. Example images of immunostaining against neuronal precursor marker nestin (*A*) and human nuclei (*B*) with neuronal marker βIII-tubulin and astrocyte marker GFAP. Our cultures at week 5 post-differentiation generate dense human nuclei-positive neuronal populations (mean ± sem % human nuclei^+^ cells with βIII-tubulin; C9–1, 96.8 ± 4.1; C9–1Δ, 97.4 ± 3.0; C9–2, 90.4 ± 3.2; C9–2Δ, 90.2 ± 4.4; C9–3, 98.5 ± 4.9; C9–3Δ, 96.4 ± 7.1; data from 3 de novo plate downs) with only negligible detectable levels of nestin (mean ± sem % nestin; C9–1, 1.1 ± 0.01; C9–1Δ, 2.5 ± 0.1; C9–2, 2.3 ± 0.1; C9–2Δ, 3.5 ± 0.1; C9–3, 1.0 ± 0.1; C9–3Δ, 2.0 ± 0.1; data from 3 de novo plate downs). Data are consistent with previous cortical neuron differentiations with cell lines used in this study and a cortical neuron protocol that gives rise to a highly efficient neuronal differentiation [6, 34, 52];. Scale bars, 100 μm.

**Additional file 2: Supplementary Figure 2.** *A, B* and C. Neuronal specification. RNA-seq analysis of established neuronal and glia markers (MAPT, NEFL, ALDH1L1, AQ4, MYRF), neuronal markers for anterior/cortical development (OTX2, PAX6, FOXP4, BCL6), hindbrain development (HOXB2), cortical layers (CUX1, POU3F2, PCP4, FOXP2), plus glutamatergic (CAMK2A, SLC17A6 and SLC17A7) and GABA-ergic neurons (GAD2, SLC32A1, PVALB) in C9 and C9-Δ lines. We note that out analysis obtained extensive detection of cortical transcripts from our cultures and are consistent with predominantly glutamatergic neurons. Note that the y axis is presented using a logarithmic scale. Data in C also show synaptic markers DLG4 and SYN1. Data are representative of mean ± sem from two pooled C9 lines (black bars) and their respective isogenic lines (red bars), as further detailed in Fig. 4. Data were derived from 3 plate downs from each line.

**Additional file 3: Supplementary Figure 3.** Pharmacological block of network activity. ***A***, Example whole cell current-clamp recordings of effect of AMPA receptor blocker, CNQX (30 μM), upon network activity. Scale bar, 50 mV, 20 s. CNQX generated full block of network burst activity. ***B***, As in A though for GABA_A_ receptor blocker, bicuculline (30 μM). Scale bar, 50 mV, 5 s. ***C***, Mean (± s.e.m.) percentage shift in burst frequency in presence of either CNQX or bicuculline for each line type (CNQX – Con, *n* = 5, *N* = 3; C9, *n* = 5, *N* = 2; C9-Δ; *n* = 5, *N* = 3 / bicuculline – Con, *n* = 5, *N* = 3; C9, *n* = 5, *N* = 3; C9-Δ; *n* = 5, *N* = 3). Bicuculline did not significantly impact upon network burst activity. Expectedly, data are consistent with an enriched population of excitatory glutamatergic cortical neurons [6, 34, 52].

**Additional file 4: Supplementary Figure 4.** Network burst data. ***A***, Mean (± s.e.m.) MEA-determined burst duration, interburst length and spike frequency within the burst for each respective *C9ORF72*^*RE*^ and *C9ORF72*^*RE*^-Δ isogenic pair (C9–1, *N* = 4; C9–1Δ, *N* = 6; C9–2, *N* = 6; C9–2Δ, *N* = 3). Significance determined by unpaired t-test. ***B***, Mean (± s.e.m.) patch-clamp-determined burst duration, interburst length and spike frequency within the burst for each respective *C9ORF72*^*RE*^ and *C9ORF72*^*RE*^-Δ isogenic pair (C9–1, *n* = 8, *N* = 2; C9–1Δ, *n* = 8, *N* = 2; C9–2, *n* = 8, *N* = 2; C9–2Δ, *n* = 5, *N* = 2). Significance determined by unpaired t-test.

**Additional file 5: Supplementary Figure 5.** Intrinsic excitability of *C9ORF72*^RE^-derived cortical neurons. ***A***, Mean (± s.e.m.) data for each Control- (*Con*), *C9ORF72*^RE^- (*C9*) and respective *C9ORF72*^RE^ gene-edited- (*C9-Δ*) derived neurons for passive membrane properties (Con, *n* = 6, *N* = 2; C9–1, *n* = 20, *N* = 3; C9–1Δ, *n* = 11, *N* = 3; C9–2, *n* = 17, *N* = 3; C9–2Δ, *n* = 12, *N* = 3; C9–3, *n* = 17, *N* = 3; C9–3Δ, *n* = 15, *N* = 3). The data shows input resistance (*R*_*IN*_), whole-cell capacitance, resting membrane potential (*RMP*). ***B***, Representative whole-cell current-clamp recordings of evoked responses to current injection (− 20 pA to + 30 pA, 0.5 s duration, 5 pA increments) for a control, *C9ORF72*^RE^- and *C9ORF72*^RE^ neuron. Cells were held at − 74 mV. Scale bar (40 mV, 100 ms). ***C***, Mean (± s.e.m.) action potential (AP) number-current relationships generated from each paired *C9ORF72*^RE^- and *C9ORF72*^RE^ gene-edited-derived neurons (*C9–1*, *C9–2*, *C9–3*) including control data. ***D***, Mean (± s.e.m.) action potential parameters (threshold, amplitude, and afterhypolarisation, AHP). AP properties were measured from the first evoked AP of the rheobasic current injection. Significance determined by unpaired t-test. We note that we did find slight, but statistically significant differences for one line in the RMP and AHP data. However, these are unlikely to be the cause of the altered network excitability because they are i) extremely modest and ii) not a conserved finding across all lines.

**Additional file 6: Supplementary Figure 6.** Synaptic puncta and neurite length. ***A***, Mean (± s.e.m.) co-localised PSD-95/Synapsin-1 puncta for each respective *C9ORF72*^*RE*^ and *C9ORF72*^*RE*^-Δ isogenic pair (C9–1, *N* = 4; C9–1Δ, *N* = 4; C9–2, *N* = 4; C9–2Δ, *N* = 4; C9–3, *N* = 4; C9–3Δ, *N* = 4). Significance determined by unpaired t-test. ***B***. To address neuronal morphology we transduced cortical NPCs with a low GFP-lentivirus titre in order to be able to visualise individual neurons. As a measure of  morphology, we then measured the neurite lengths (total sum of all processes) for each cell. ***C***. Data show the mean ± s.e.m. neurite length (in μm) for each respective *C9ORF72*^*RE*^ and *C9ORF72*^*RE*^-Δ isogenic pair (C9–1, *n* = 44 cells; C9–1Δ, *n* = 24; C9–2, *n* = 39; C9–2Δ, *n* = 20; C9–3, *n* = 17; C9–3Δ, *n* = 27). All data derived from 2 de novo preparations. Significance determined by unpaired t-test.

**Additional file 7: Supplementary Figure 7.** mEPSC amplitude, rise time and decay properties. ***A***, Cumulative probability plots of mEPSC inter-event time for each *C9ORF72*^RE^- and corresponding *C9ORF72*^RE^ gene-edited neurons. Data was obtained from at least 2-min recordings and neurons that displayed mEPSC frequencies under 0.05 Hz were omitted from the analysis. Significance of cumulative probability plots determined using Kolmogorov-Smirnov test. Mean ± s.e.m. mEPSC frequency for each line and respective edit are shown inset (C9–1, *n* = 23, *N* = 5; C9–1Δ, *n* = 15, *N* = 4; C9–2, *n* = 17, *N* = 4; C9–2Δ, *n* = 11, *N* = 3; C9–3, *n* = 12, *N* = 3; C9–3Δ, *n* = 16, *N* = 4). ***B***, As **A** though for mEPSC amplitude. ***C***, Mean ± s.e.m. mEPSC rise time (10–90%) and τ decay properties for each line (C9–1, *n* = 15, *N* = 4; C9–1Δ, *n* = 12, *N* = 3; C9–2, *n* = 12, *N* = 3; C9–2Δ, *n* = 5, *N* = 2; C9–3, *n* = 8, N = 3; C9–3Δ, *n* = 13, *N* = 3). Significance determined by Welch’s t-test or unpaired t-test. Other than mEPSC frequency (Fig. 2), data are not consistent with altered mEPSC properties. We note that we did find slight, but statistically significant differences for one line in the mEPSC amplitude and rise time data. However, these are unlikely to be the cause of altered network excitability observed in our cultures because they are i) extremely modest and ii) not a conserved finding across all lines. ***D.*** RNA-seq analysis of AMPA receptor subunits (GRIA1–4) in C9 and C9-Δ lines. Note that the y axis is presented using a logarithmic scale. Data are representative of mean ± sem from two pooled C9 lines (*black bars*) and their respective isogenic lines (*red bars*), as detailed in Fig. 4. Data were derived from 3 plate downs from each line. The data are not consistent with any change in expression between C9 and C9-Δ lines.

**Additional file 8: Supplementary Figure 8.** mEPSC amplitude in the presence and absence of hypertonic sucrose. ***A*,** Representative recording of mEPSC activity before (baseline) and in the presence of sucrose (0.5 M, *filled bar*). The initial transient and steady-state phases of the sucrose-evoked response are highlighted. Scale bars; 50 pA, 5 s. ***B*,** Mean ± s.e.m. fold change in mEPSC frequency for each line for the transient (C9–1, *n* = 9, N = 3; C9–1Δ, *n* = 10, N = 3; C9–2, n = 8, N = 3; C9–2Δ, *n* = 6, N = 2; C9–3, n = 11, N = 4; C9–3Δ, *n* = 14, N = 4) and steady state phases (C9–1, *n* = 7, N = 2; C9–1Δ, n = 6, N = 3; C9–2, n = 8, N = 3; C9–2Δ, n = 6, N = 2; C9–3, n = 8, N = 3; C9–3Δ, n = 11, N = 3). ***C***, As in ***B***, though for mEPSC amplitude for the transient (C9–1, n = 7; C9–1Δ, n = 6; C9–2, n = 8; C9–2Δ, n = 6; C9–3, n = 10, N = 3; C9–3Δ, n = 12, N = 3) and steady-state phases (C9–1, n = 7; C9–1Δ, n = 6; C9–2, n = 8; C9–2Δ, n = 6; C9–3, n = 7, N = 3; C9–3Δ, n = 11, N = 3). Significance determined by two-tailed unpaired t-test. The patch pipette solution was supplemented with BAPTA (1 mM) to prevent potential Ca^2+^-dependent modulation of post-synaptic neuron properties.

**Additional file 9: Supplementary Figure 9.** KCl-evoked release properties. ***A***, Sample traces from recordings of mEPSC events before and in the presence of KCl (30 mM) from *C9ORF72*^RE^- and *C9ORF72*^RE^-Δ-cortical neurons (C9–2 and C9–2Δ). Scale bars; 50 pA, 5 s. ***B***, Mean ± s.e.m. fold change in mEPSC frequency for each line in the presence of KCl (C9–1, *n* = 5, N = 2; C9–1Δ, n = 5, N = 2; C9–2, n = 3, N = 2; C9–2Δ, n = 3, N = 2; C9–3, N = 2, n = 7, C9–3Δ, n = 5, N = 2). No statistical difference between each *C9ORF72*^*RE*^ and *C9ORF72*^*RE*^-Δ pair was determined (unpaired t-test). mEPSC frequency in the presence of KCl was determined from a stretch of recording at least 1 min in duration. The patch pipette solution was supplemented with BAPTA (1 mM) to prevent potential Ca^2+^-dependent modulation of post-synaptic neuron properties.

**Additional file 10: Supplementary Figure 10.** Depolarisation-mediated mEPSC amplitude potentiation. ***A***. Example recordings of mEPSCs prior (1. *light blue*) to and after (2. *blue*) the depolarisation pulse protocol (DPP, 10 depolarising pulses of 3 s in duration, every 9 s, from − 84 to + 16 mV). Example post-DPP mEPSCs are sampled from the 8–10 min stretch of data. Scale bar; 10 pA, 2.5 s. ***B***. Individual mEPSC amplitude plot in an example experiment. mEPSCs before (1) and after DPP (2) are represented in *light blue* and *blue*, respectively. The *grey bar* indicates the stimulation period. Note the transient increase in mEPSC amplitude post-DPP. ***C***. Cumulative probability plot showing a shift (*p* < 0.001, Kolmogorov–Smirnov test) in mEPSC amplitude data in B from the initial control period (1. *light blue*) to the 10 min post-DPP period (2. *blue*) in which there is consistent, transient potentiation of mEPSC amplitude. ***D***. *Left*, mean mEPSCs for data shown in B for initial control period (1. *light blue*) and 10 min post-DPP (2. *blue*). Scale bar; 5 pA, 5 ms. *Right*, mean mEPSCs scaled to amplitude and time base. ***E***. To test whether potentiation was Ca^2+^-dependent we performed DPP in the presence of nifedipine, a blocker of voltage-gated Ca^2+^ channels, or BAPTA, a Ca^2+^ chelator, supplemented to the patch pipette. Data shows mean ± s.e.m. fold increase of mEPSC amplitude 10 min post-DPP from initial control period for the control line (*n* = 17), + nifedipine (n = 14) and + BAPTA (n = 6). Example traces and data presented in Supplementary Figure 11. Significance determined by one-way ANOVA with Bonferroni’s post hoc test.

**Additional file 11: Supplementary Figure 11.** Ca^2+^-dependent mEPSC potentiation. **A**. Example recordings of mEPSCs prior to (1. *light blue*) and after (2. *blue*) depolarisation in the presence of nifedipine (10 μM, applied to the extracellular solution). Scale bar; 20 pA, 2.5 s. **B**. Individual mEPSC amplitude plots for DPP experiments in the presence of nifedipine. mEPSCs before (1) and after DPP (2) are represented in *light blue* and *blue*, respectively. **C**. Cumulative probability plot of mEPSC amplitudes for the initial control period (1. *light blue*) and after DPP (2. *blue*) in the presence of nifedipine of the data shown in B. Whilst shift in mEPSC amplitude in the presence of nifedipine is significant (*p* < 0.01, Kolmogorov–Smirnov test), the shift is a decrease in mEPSC amplitude. **D**. As in A though in the presence of BAPTA (10 mM, to the intracellular solution). **E**. As in B though in the presence of BAPTA. **F**. Cumulative probability plot of mEPSC amplitudes for the initial control period (1. *light blue*) and after DPP (2. *blue*) in the presence of BAPTA for the data shown in E. Shift in mESPC amplitude is not significant (*p* = 0.937, Kolmogorov–Smirnov test). **G**. The fold change in mEPSC decay time constant post-DPP with respect to baseline activity (Con, n = 5; C9, n = 5; C9-Δ, n = 5). Fold changes are not significant (one-way ANOVA with Bonferroni’s multiple comparisons test). Data are consistent with the fact that average scaled mEPSC traces from pre- and post-DPP stages are superimposable.

**Additional file 12.**

## Data Availability

The datasets used and/or analysed during the current study are available from the corresponding author on reasonable request.

## Abbreviations

ALS: Amyotrophic lateral sclerosis
AMPA: (α-amino-3-hydroxy-5-methyl-4-isoxazolepropionic acid)
ANOVA: Analysis of variance
aNPC: anterior neural precursors
*C9ORF72*^*RE*^: C9ORF72 repeat expansion
CNQX: Cyanquixaline (6-cyano-7-nitroquinoxaline-2,3-dione)
DPP: Depolarisation pulse protocol
FTD: Frontotemporal dementia
GABA: Gamma aminobutyric acid
GO: Gene ontology
iPSC: induced pluripotent stem cell
MEA: Multi-electrode array
mEPSC: mini excitatory post-synaptic current
RNA: Ribonucleic acid
RRP: Readily releasable pool
RT: Room temperature
s.e.m.: standard error of the mean
