# Supplementary figures and images for: Altered network properties in C9ORF72 repeat expansion cortical neurons are due to synaptic dysfunction
Source: Mol Neurodegener. 2021 Mar 4;16:13. doi: 10.1186/s13024-021-00433-8 (PMC7931347; doi:10.1186/s13024-021-00433-8)

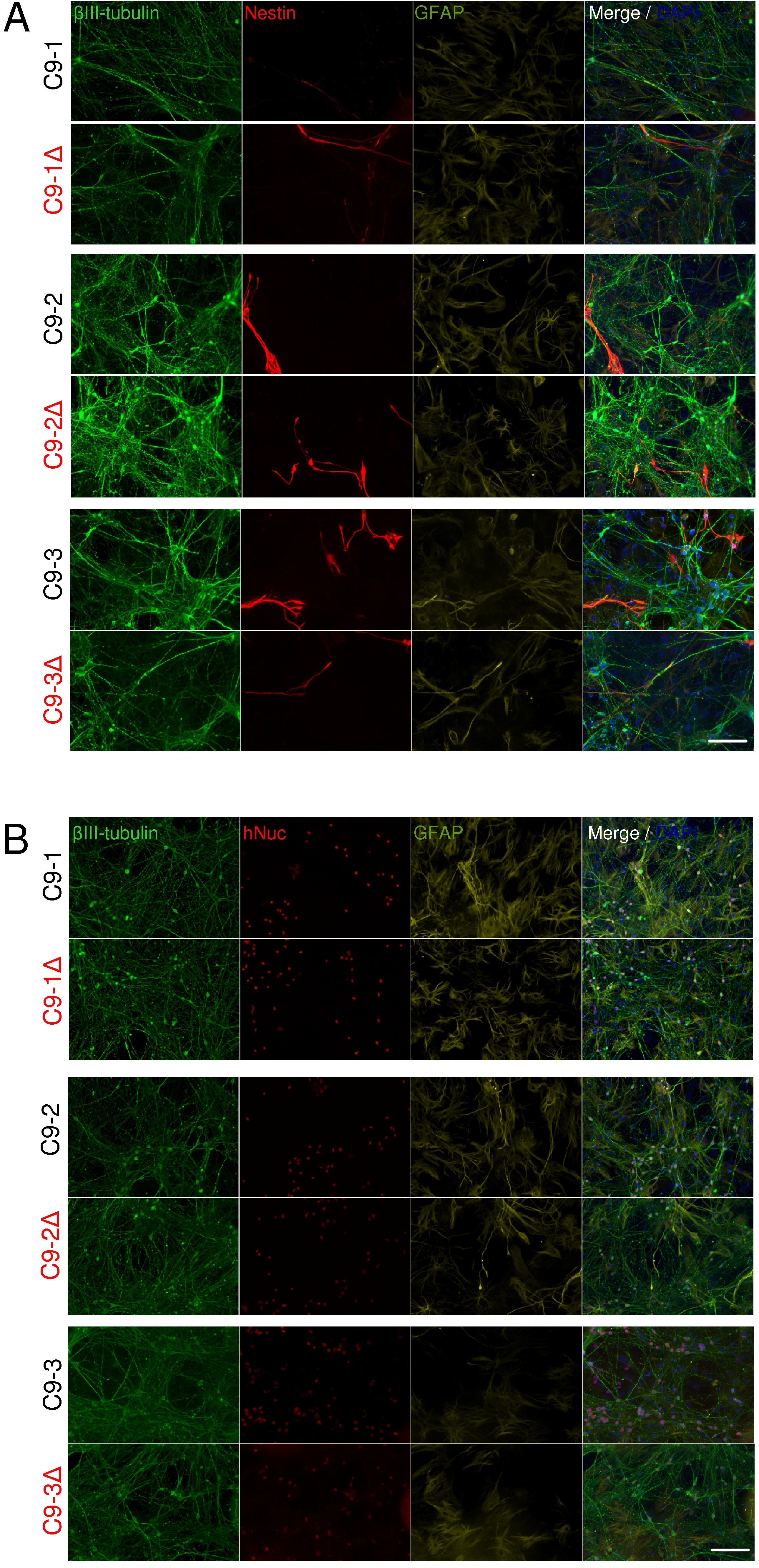

Supplement: Supplementary file 1 — Additional file 1: Supplementary Figure 1. Neuronal specification. Example images of immunostaining against neuronal precursor marker nestin (A) and human nuclei (B) with neuronal marker βIII-tubulin and astrocyte marker GFAP. Our cultures at week 5 post-differentiation generate dense human nuclei-positive neuronal populations (mean ± sem % human nuclei+ cells with βIII-tubulin; C9–1, 96.8 ± 4.1; C9–1Δ, 97.4 ± 3.0; C9–2, 90.4 ± 3.2; C9–2Δ, 90.2 ± 4.4; C9–3, 98.5 ± 4.9; C9–3Δ, 96.4 ± 7.1; data from 3 de novo plate downs) with only negligible detectable levels of nestin (mean ± sem % nestin; C9–1, 1.1 ± 0.01; C9–1Δ, 2.5 ± 0.1; C9–2, 2.3 ± 0.1; C9–2Δ, 3.5 ± 0.1; C9–3, 1.0 ± 0.1; C9–3Δ, 2.0 ± 0.1; data from 3 de novo plate downs). Data are consistent with previous cortical neuron differentiations with cell lines used in this study and a cortical neuron protocol that gives rise to a highly efficient neuronal differentiation [6, 34, 52];. Scale bars, 100 μm. [file 13024_2021_433_MOESM1_ESM.tif]

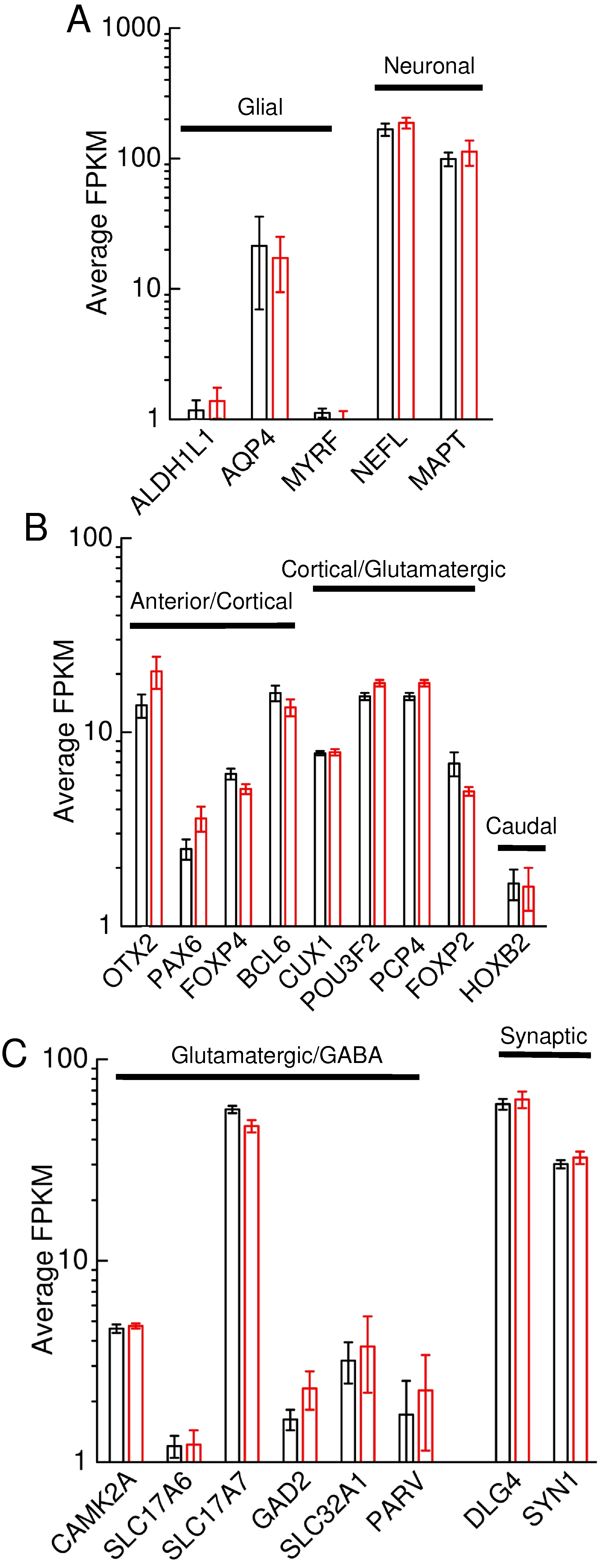

Supplement: Supplementary file 2 — Additional file 2: Supplementary Figure 2. A, B and C. Neuronal specification. RNA-seq analysis of established neuronal and glia markers (MAPT, NEFL, ALDH1L1, AQ4, MYRF), neuronal markers for anterior/cortical development (OTX2, PAX6, FOXP4, BCL6), hindbrain development (HOXB2), cortical layers (CUX1, POU3F2, PCP4, FOXP2), plus glutamatergic (CAMK2A, SLC17A6 and SLC17A7) and GABA-ergic neurons (GAD2, SLC32A1, PVALB) in C9 and C9-Δ lines. We note that out analysis obtained extensive detection of cortical transcripts from our cultures and are consistent with predominantly glutamatergic neurons. Note that the y axis is presented using a logarithmic scale. Data in C also show synaptic markers DLG4 and SYN1. Data are representative of mean ± sem from two pooled C9 lines (black bars) and their respective isogenic lines (red bars), as further detailed in Fig. 4. Data were derived from 3 plate downs from each line. [file 13024_2021_433_MOESM2_ESM.tif]

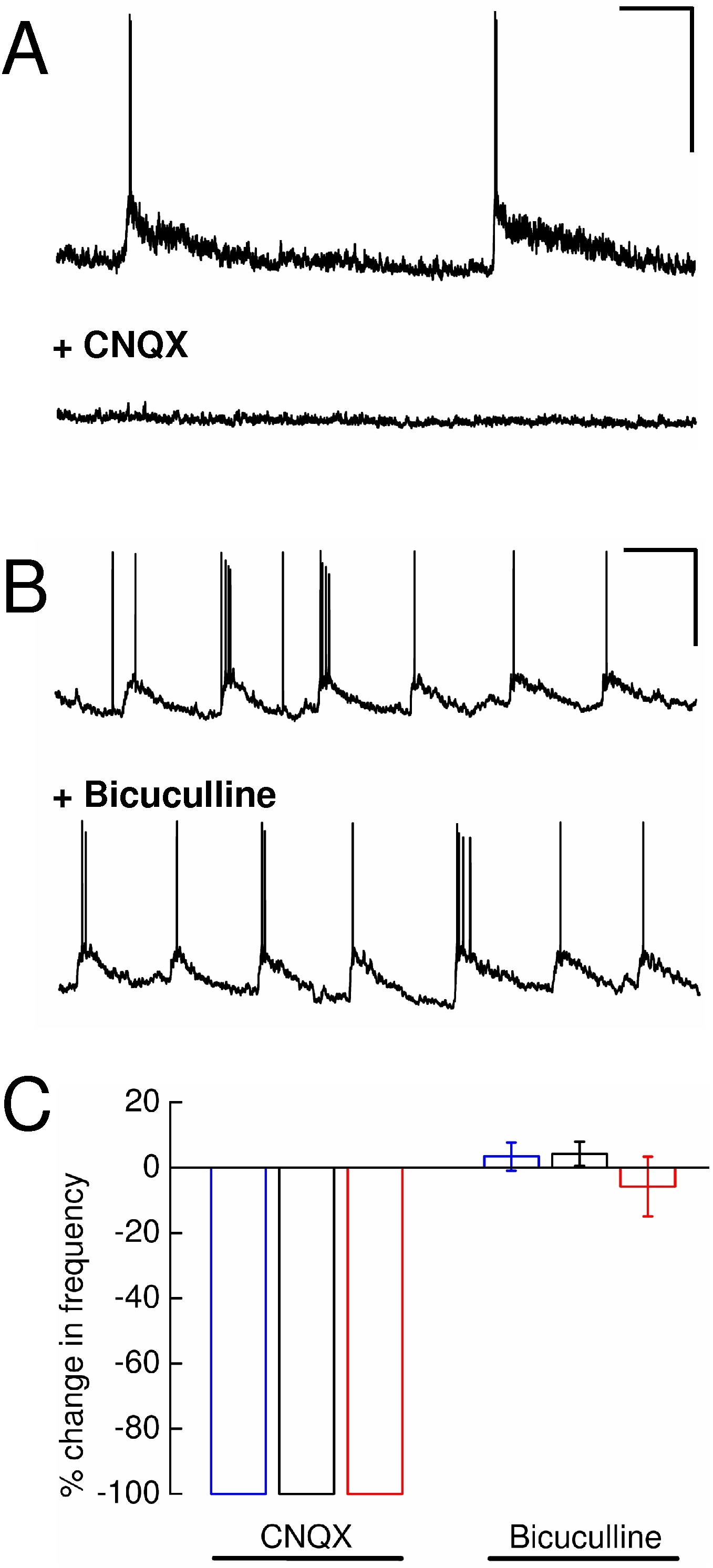

Supplement: Supplementary file 3 — Additional file 3: Supplementary Figure 3. Pharmacological block of network activity. A, Example whole cell current-clamp recordings of effect of AMPA receptor blocker, CNQX (30 μM), upon network activity. Scale bar, 50 mV, 20 s. CNQX generated full block of network burst activity. B, As in A though for GABAA receptor blocker, bicuculline (30 μM). Scale bar, 50 mV, 5 s. C, Mean (± s.e.m.) percentage shift in burst frequency in presence of either CNQX or bicuculline for each line type (CNQX – Con, n = 5, N = 3; C9, n = 5, N = 2; C9-Δ; n = 5, N = 3 / bicuculline – Con, n = 5, N = 3; C9, n = 5, N = 3; C9-Δ; n = 5, N = 3). Bicuculline did not significantly impact upon network burst activity. Expectedly, data are consistent with an enriched population of excitatory glutamatergic cortical neurons [6, 34, 52]. [file 13024_2021_433_MOESM3_ESM.tif]

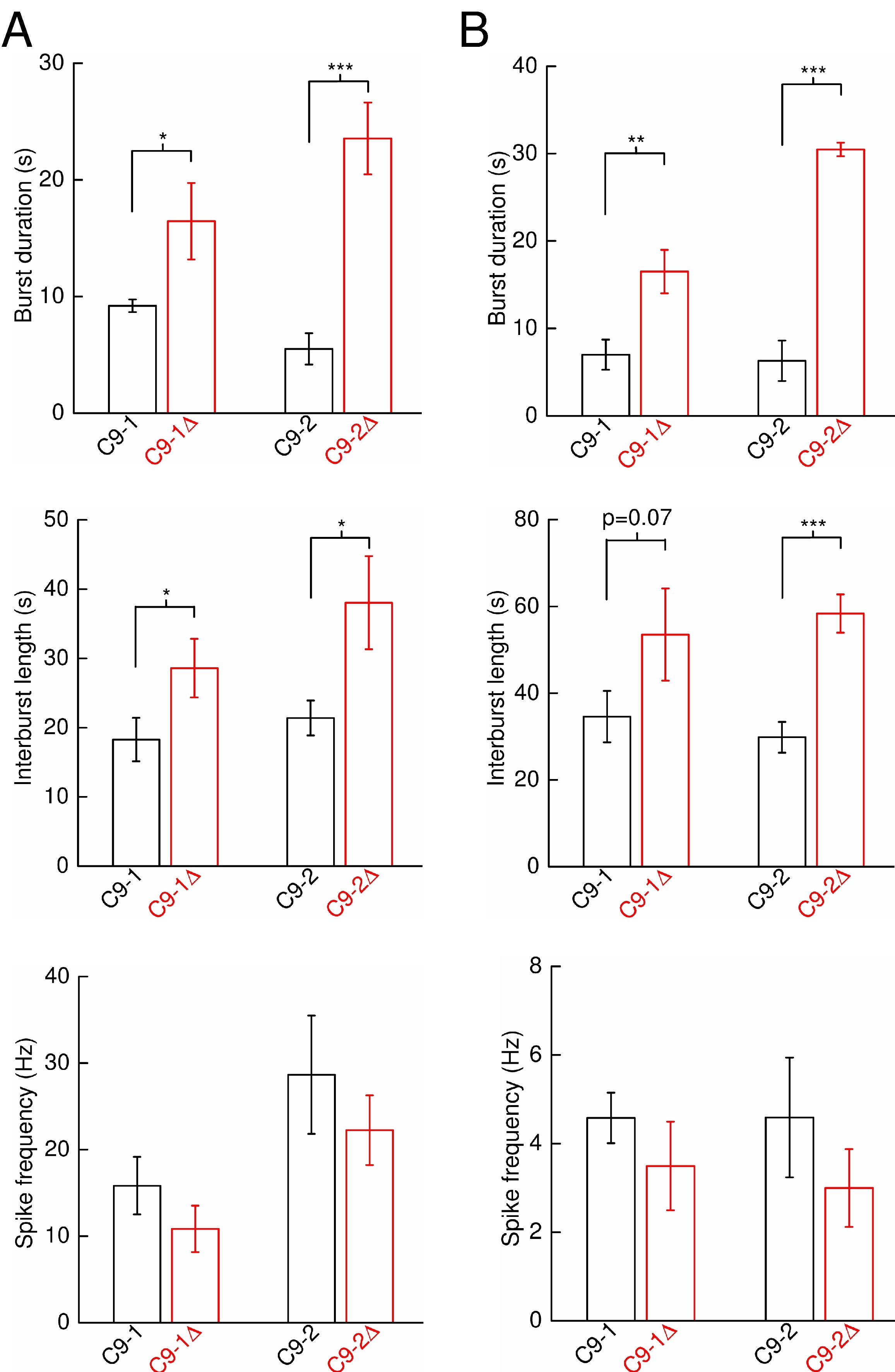

Supplement: Supplementary file 4 — Additional file 4: Supplementary Figure 4. Network burst data. A, Mean (± s.e.m.) MEA-determined burst duration, interburst length and spike frequency within the burst for each respective C9ORF72RE and C9ORF72RE-Δ isogenic pair (C9–1, N = 4; C9–1Δ, N = 6; C9–2, N = 6; C9–2Δ, N = 3). Significance determined by unpaired t-test. B, Mean (± s.e.m.) patch-clamp-determined burst duration, interburst length and spike frequency within the burst for each respective C9ORF72RE and C9ORF72RE-Δ isogenic pair (C9–1, n = 8, N = 2; C9–1Δ, n = 8, N = 2; C9–2, n = 8, N = 2; C9–2Δ, n = 5, N = 2). Significance determined by unpaired t-test. [file 13024_2021_433_MOESM4_ESM.tif]

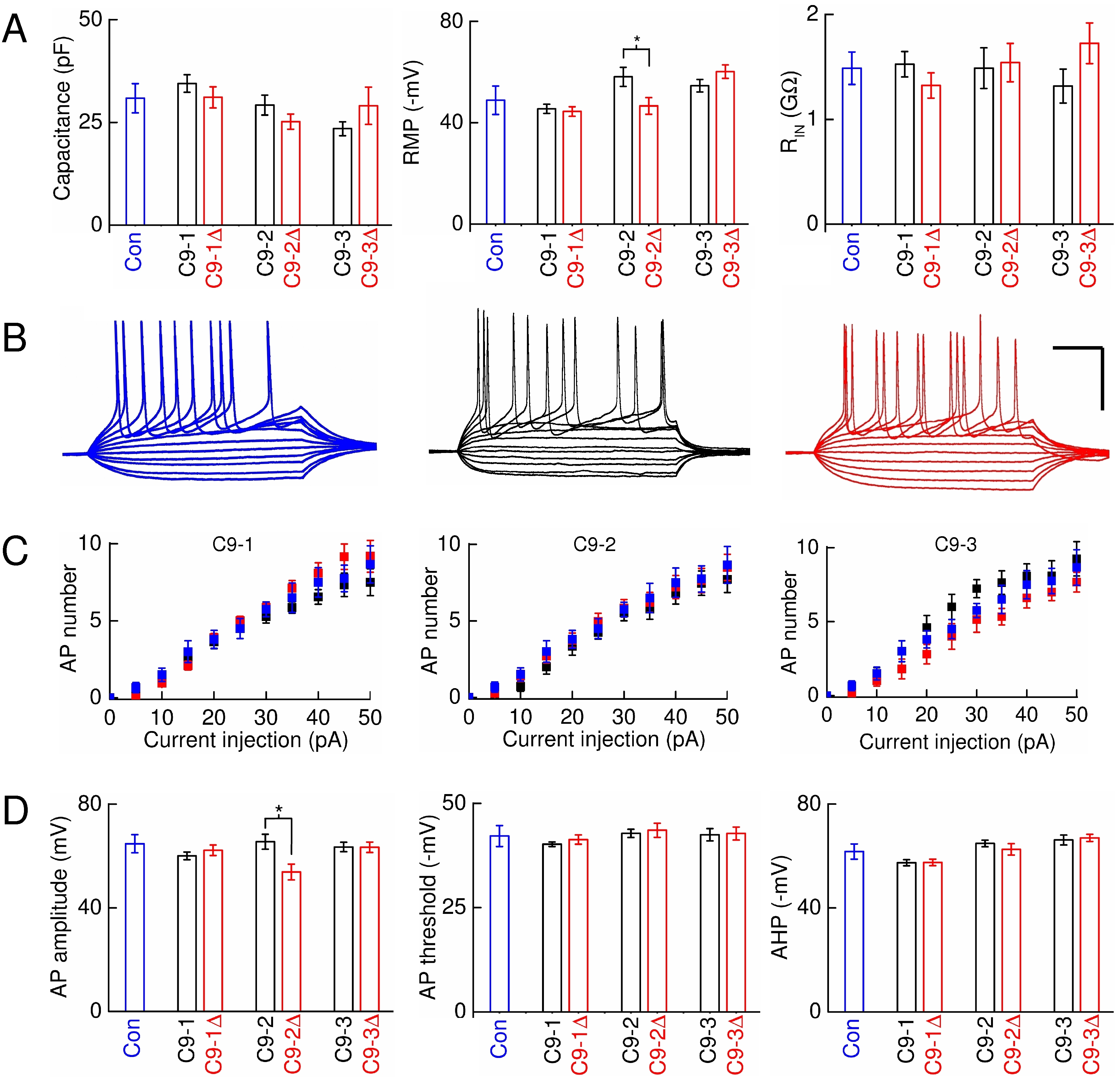

Supplement: Supplementary file 5 — Additional file 5: Supplementary Figure 5. Intrinsic excitability of C9ORF72RE-derived cortical neurons. A, Mean (± s.e.m.) data for each Control- (Con), C9ORF72RE- (C9) and respective C9ORF72RE gene-edited- (C9-Δ) derived neurons for passive membrane properties (Con, n = 6, N = 2; C9–1, n = 20, N = 3; C9–1Δ, n = 11, N = 3; C9–2, n = 17, N = 3; C9–2Δ, n = 12, N = 3; C9–3, n = 17, N = 3; C9–3Δ, n = 15, N = 3). The data shows input resistance (RIN), whole-cell capacitance, resting membrane potential (RMP). B, Representative whole-cell current-clamp recordings of evoked responses to current injection (− 20 pA to + 30 pA, 0.5 s duration, 5 pA increments) for a control, C9ORF72RE- and C9ORF72RE neuron. Cells were held at − 74 mV. Scale bar (40 mV, 100 ms). C, Mean (± s.e.m.) action potential (AP) number-current relationships generated from each paired C9ORF72RE- and C9ORF72RE gene-edited-derived neurons (C9–1, C9–2, C9–3) including control data. D, Mean (± s.e.m.) action potential parameters (threshold, amplitude, and afterhypolarisation, AHP). AP properties were measured from the first evoked AP of the rheobasic current injection. Significance determined by unpaired t-test. We note that we did find slight, but statistically significant differences for one line in the RMP and AHP data. However, these are unlikely to be the cause of the altered network excitability because they are i) extremely modest and ii) not a conserved finding across all lines. [file 13024_2021_433_MOESM5_ESM.tif]

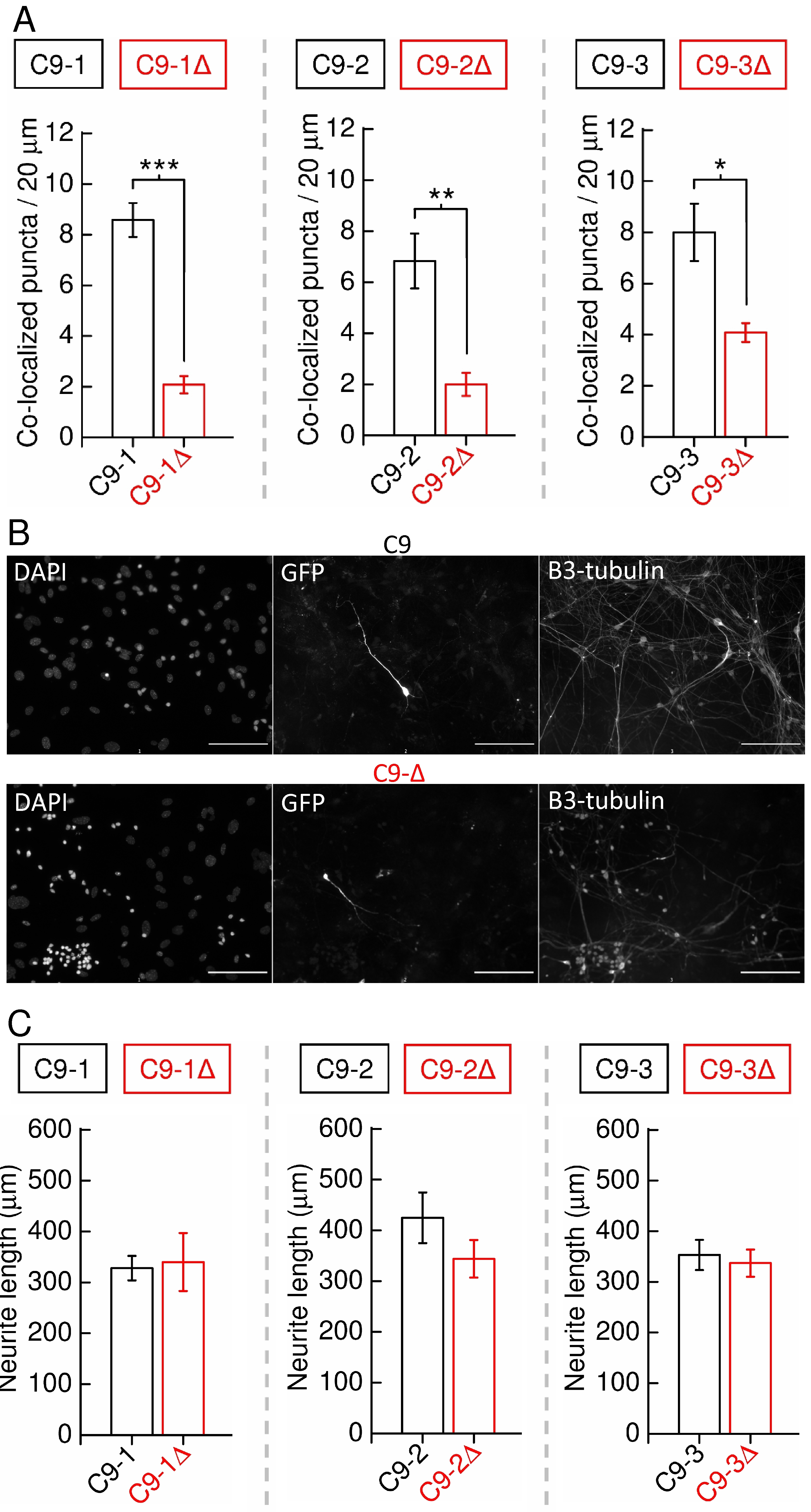

Supplement: Supplementary file 6 — Additional file 6: Supplementary Figure 6. Synaptic puncta and neurite length. A, Mean (± s.e.m.) co-localised PSD-95/Synapsin-1 puncta for each respective C9ORF72RE and C9ORF72RE-Δ isogenic pair (C9–1, N = 4; C9–1Δ, N = 4; C9–2, N = 4; C9–2Δ, N = 4; C9–3, N = 4; C9–3Δ, N = 4). Significance determined by unpaired t-test. B. To address neuronal morphology we transduced cortical NPCs with a low GFP-lentivirus titre in order to be able to visualise individual neurons. As a measure of morphology, we then measured the neurite lengths (total sum of all processes) for each cell. C. Data show the mean ± s.e.m. neurite length (in μm) for each respective C9ORF72RE and C9ORF72RE-Δ isogenic pair (C9–1, n = 44 cells; C9–1Δ, n = 24; C9–2, n = 39; C9–2Δ, n = 20; C9–3, n = 17; C9–3Δ, n = 27). All data derived from 2 de novo preparations. Significance determined by unpaired t-test. [file 13024_2021_433_MOESM6_ESM.tif]

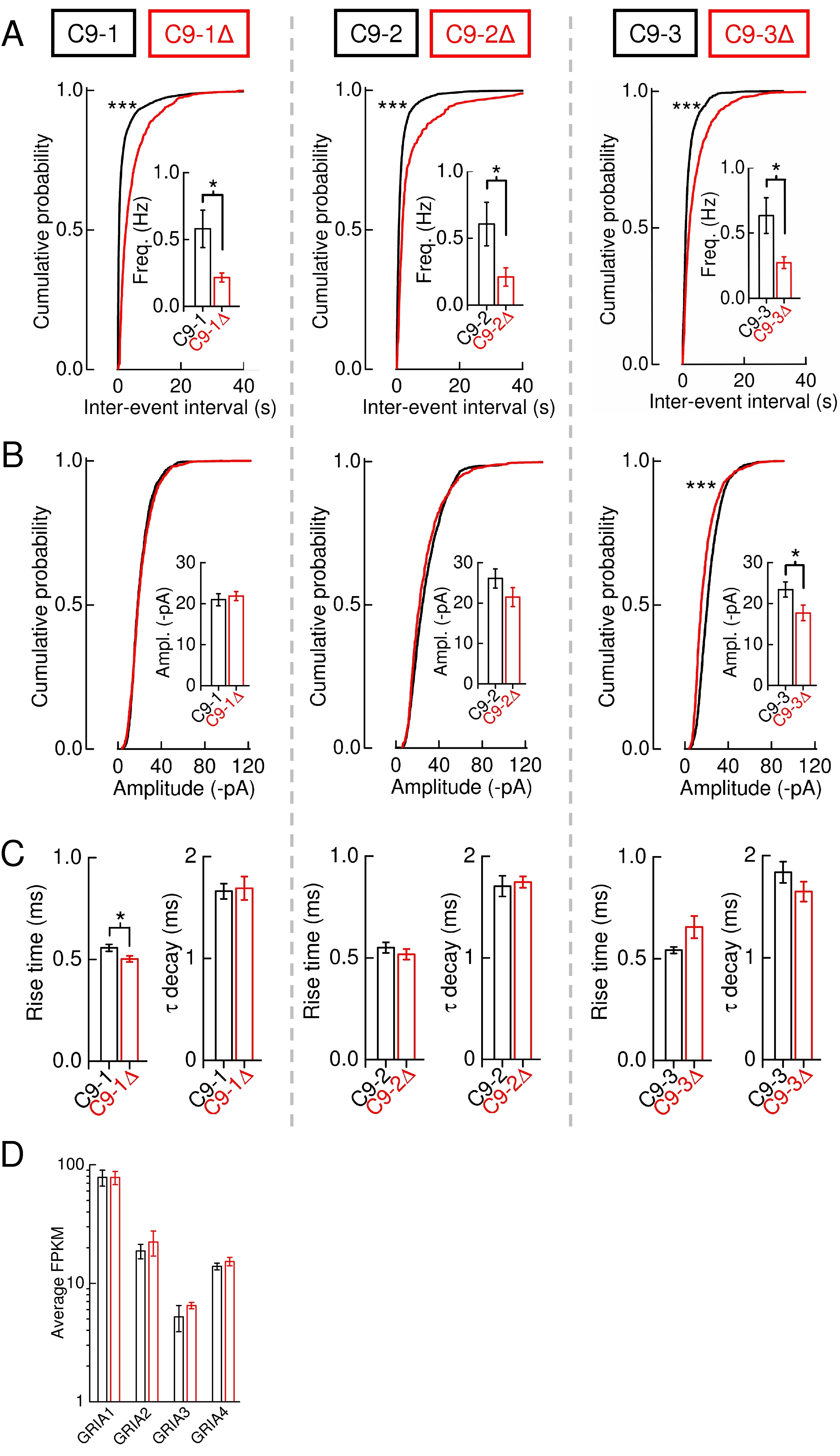

Supplement: Supplementary file 7 — Additional file 7: Supplementary Figure 7. mEPSC amplitude, rise time and decay properties. A, Cumulative probability plots of mEPSC inter-event time for each C9ORF72RE- and corresponding C9ORF72RE gene-edited neurons. Data was obtained from at least 2-min recordings and neurons that displayed mEPSC frequencies under 0.05 Hz were omitted from the analysis. Significance of cumulative probability plots determined using Kolmogorov-Smirnov test. Mean ± s.e.m. mEPSC frequency for each line and respective edit are shown inset (C9–1, n = 23, N = 5; C9–1Δ, n = 15, N = 4; C9–2, n = 17, N = 4; C9–2Δ, n = 11, N = 3; C9–3, n = 12, N = 3; C9–3Δ, n = 16, N = 4). B, As A though for mEPSC amplitude. C, Mean ± s.e.m. mEPSC rise time (10–90%) and τ decay properties for each line (C9–1, n = 15, N = 4; C9–1Δ, n = 12, N = 3; C9–2, n = 12, N = 3; C9–2Δ, n = 5, N = 2; C9–3, n = 8, N = 3; C9–3Δ, n = 13, N = 3). Significance determined by Welch’s t-test or unpaired t-test. Other than mEPSC frequency (Fig. 2), data are not consistent with altered mEPSC properties. We note that we did find slight, but statistically significant differences for one line in the mEPSC amplitude and rise time data. However, these are unlikely to be the cause of altered network excitability observed in our cultures because they are i) extremely modest and ii) not a conserved finding across all lines. D. RNA-seq analysis of AMPA receptor subunits (GRIA1–4) in C9 and C9-Δ lines. Note that the y axis is presented using a logarithmic scale. Data are representative of mean ± sem from two pooled C9 lines (black bars) and their respective isogenic lines (red bars), as detailed in Fig. 4. Data were derived from 3 plate downs from each line. The data are not consistent with any change in expression between C9 and C9-Δ lines. [file 13024_2021_433_MOESM7_ESM.tif]

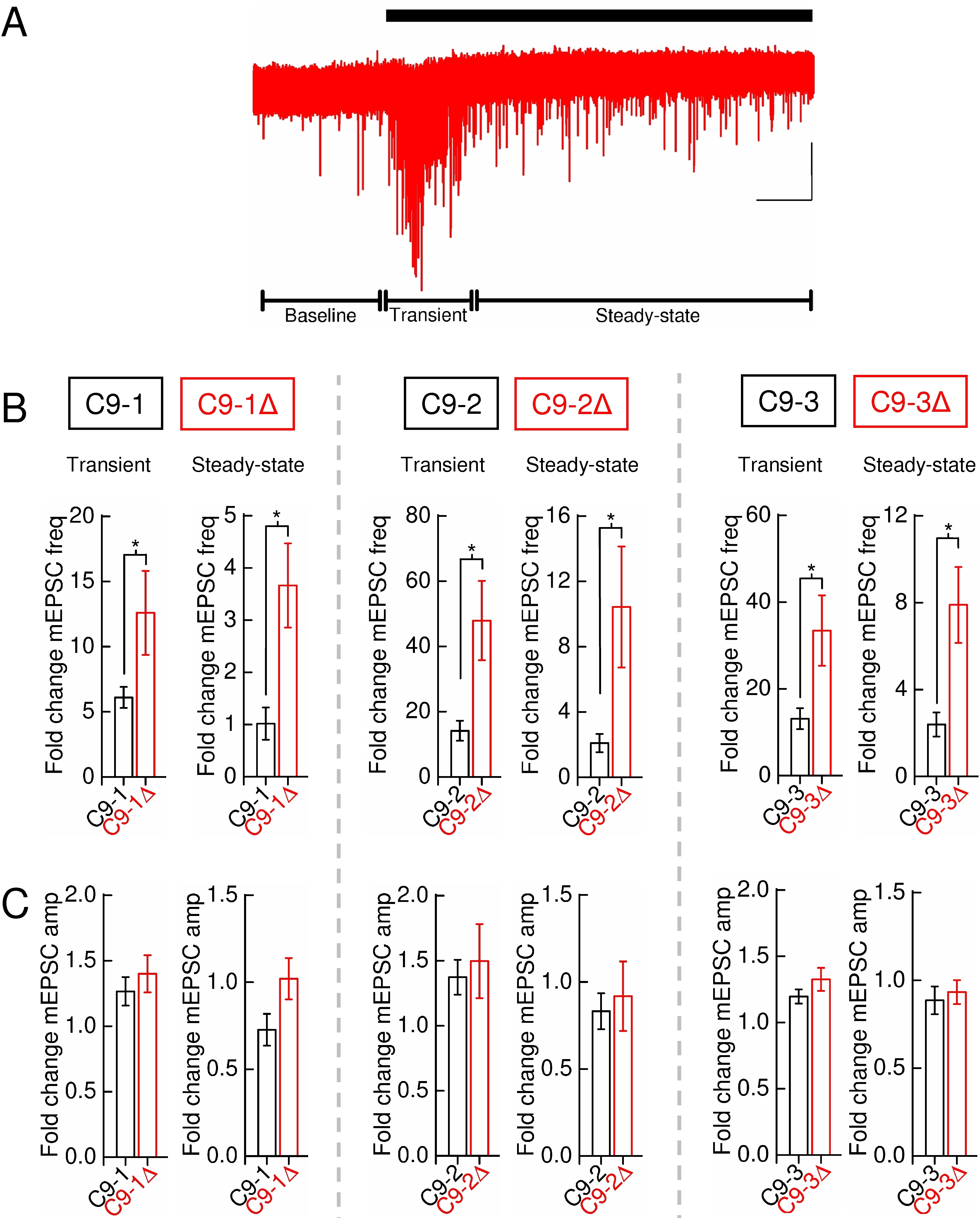

Supplement: Supplementary file 8 — Additional file 8: Supplementary Figure 8. mEPSC amplitude in the presence and absence of hypertonic sucrose. A, Representative recording of mEPSC activity before (baseline) and in the presence of sucrose (0.5 M, filled bar). The initial transient and steady-state phases of the sucrose-evoked response are highlighted. Scale bars; 50 pA, 5 s. B, Mean ± s.e.m. fold change in mEPSC frequency for each line for the transient (C9–1, n = 9, N = 3; C9–1Δ, n = 10, N = 3; C9–2, n = 8, N = 3; C9–2Δ, n = 6, N = 2; C9–3, n = 11, N = 4; C9–3Δ, n = 14, N = 4) and steady state phases (C9–1, n = 7, N = 2; C9–1Δ, n = 6, N = 3; C9–2, n = 8, N = 3; C9–2Δ, n = 6, N = 2; C9–3, n = 8, N = 3; C9–3Δ, n = 11, N = 3). C, As in B, though for mEPSC amplitude for the transient (C9–1, n = 7; C9–1Δ, n = 6; C9–2, n = 8; C9–2Δ, n = 6; C9–3, n = 10, N = 3; C9–3Δ, n = 12, N = 3) and steady-state phases (C9–1, n = 7; C9–1Δ, n = 6; C9–2, n = 8; C9–2Δ, n = 6; C9–3, n = 7, N = 3; C9–3Δ, n = 11, N = 3). Significance determined by two-tailed unpaired t-test. The patch pipette solution was supplemented with BAPTA (1 mM) to prevent potential Ca2+-dependent modulation of post-synaptic neuron properties. [file 13024_2021_433_MOESM8_ESM.tif]

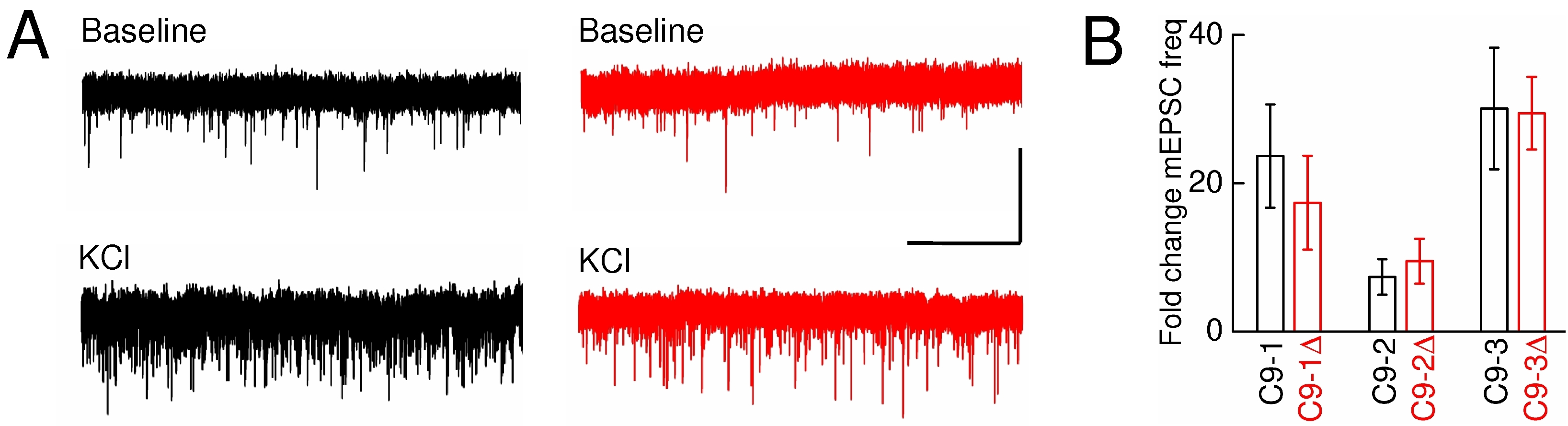

Supplement: Supplementary file 9 — Additional file 9: Supplementary Figure 9. KCl-evoked release properties. A, Sample traces from recordings of mEPSC events before and in the presence of KCl (30 mM) from C9ORF72RE- and C9ORF72RE-Δ-cortical neurons (C9–2 and C9–2Δ). Scale bars; 50 pA, 5 s. B, Mean ± s.e.m. fold change in mEPSC frequency for each line in the presence of KCl (C9–1, n = 5, N = 2; C9–1Δ, n = 5, N = 2; C9–2, n = 3, N = 2; C9–2Δ, n = 3, N = 2; C9–3, N = 2, n = 7, C9–3Δ, n = 5, N = 2). No statistical difference between each C9ORF72RE and C9ORF72RE-Δ pair was determined (unpaired t-test). mEPSC frequency in the presence of KCl was determined from a stretch of recording at least 1 min in duration. The patch pipette solution was supplemented with BAPTA (1 mM) to prevent potential Ca2+-dependent modulation of post-synaptic neuron properties. [file 13024_2021_433_MOESM9_ESM.tif]

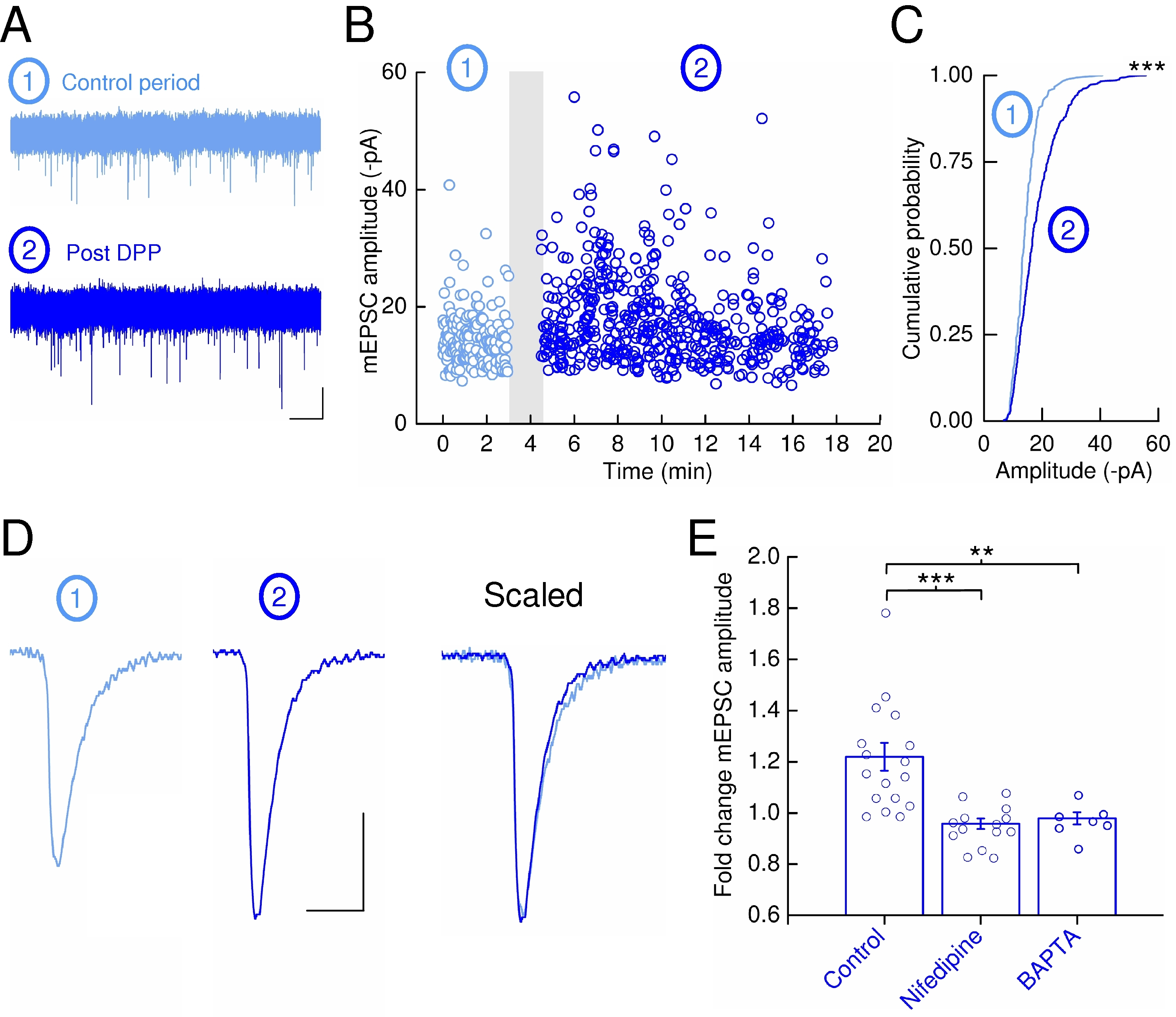

Supplement: Supplementary file 10 — Additional file 10: Supplementary Figure 10. Depolarisation-mediated mEPSC amplitude potentiation. A. Example recordings of mEPSCs prior (1. light blue) to and after (2. blue) the depolarisation pulse protocol (DPP, 10 depolarising pulses of 3 s in duration, every 9 s, from − 84 to + 16 mV). Example post-DPP mEPSCs are sampled from the 8–10 min stretch of data. Scale bar; 10 pA, 2.5 s. B. Individual mEPSC amplitude plot in an example experiment. mEPSCs before (1) and after DPP (2) are represented in light blue and blue, respectively. The grey bar indicates the stimulation period. Note the transient increase in mEPSC amplitude post-DPP. C. Cumulative probability plot showing a shift (p < 0.001, Kolmogorov–Smirnov test) in mEPSC amplitude data in B from the initial control period (1. light blue) to the 10 min post-DPP period (2. blue) in which there is consistent, transient potentiation of mEPSC amplitude. D. Left, mean mEPSCs for data shown in B for initial control period (1. light blue) and 10 min post-DPP (2. blue). Scale bar; 5 pA, 5 ms. Right, mean mEPSCs scaled to amplitude and time base. E. To test whether potentiation was Ca2+-dependent we performed DPP in the presence of nifedipine, a blocker of voltage-gated Ca2+ channels, or BAPTA, a Ca2+ chelator, supplemented to the patch pipette. Data shows mean ± s.e.m. fold increase of mEPSC amplitude 10 min post-DPP from initial control period for the control line (n = 17), + nifedipine (n = 14) and + BAPTA (n = 6). Example traces and data presented in Supplementary Figure 11. Significance determined by one-way ANOVA with Bonferroni’s post hoc test. [file 13024_2021_433_MOESM10_ESM.tif]

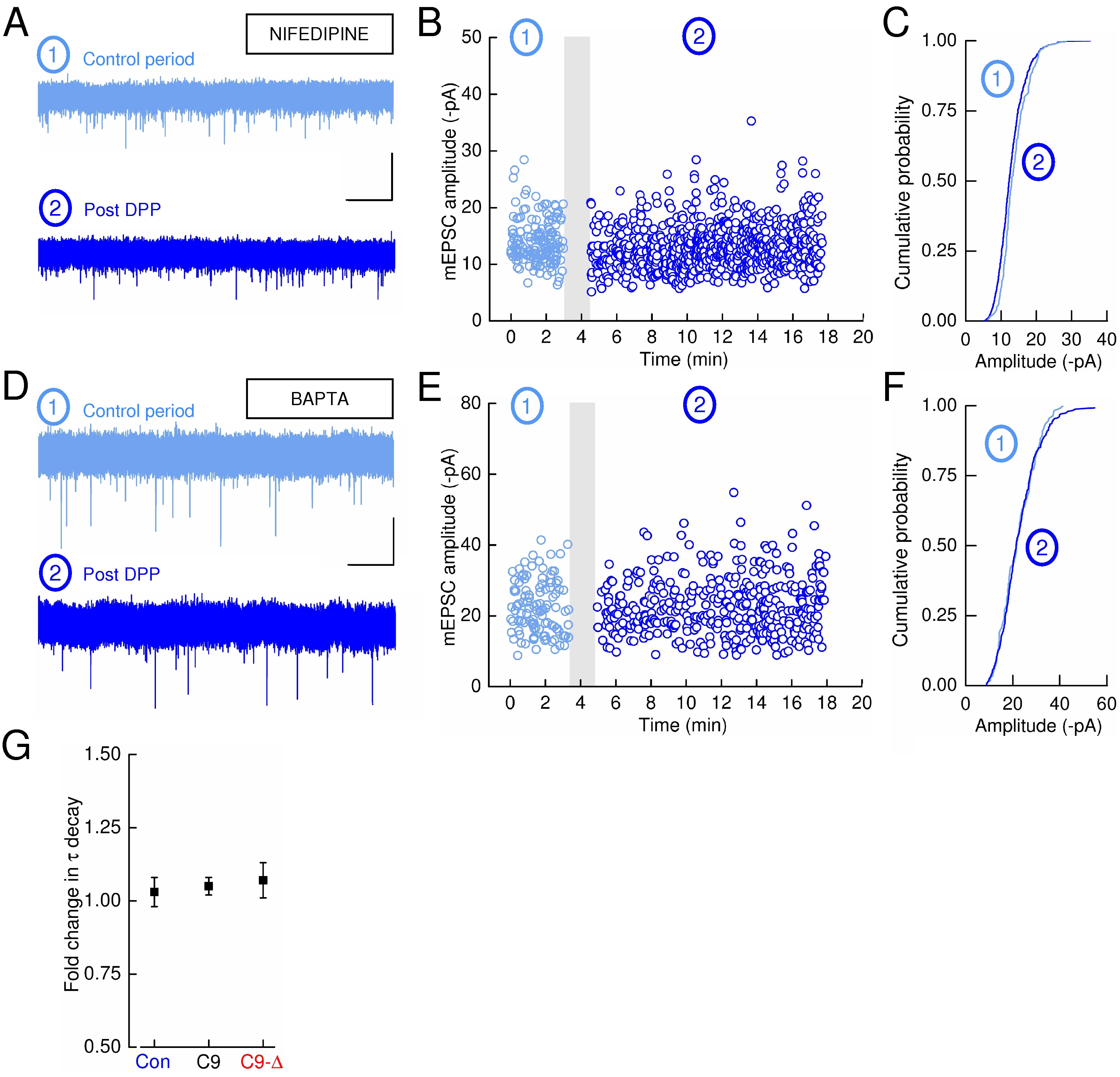

Supplement: Supplementary file 11 — Additional file 11: Supplementary Figure 11. Ca2+-dependent mEPSC potentiation. A. Example recordings of mEPSCs prior to (1. light blue) and after (2. blue) depolarisation in the presence of nifedipine (10 μM, applied to the extracellular solution). Scale bar; 20 pA, 2.5 s. B. Individual mEPSC amplitude plots for DPP experiments in the presence of nifedipine. mEPSCs before (1) and after DPP (2) are represented in light blue and blue, respectively. C. Cumulative probability plot of mEPSC amplitudes for the initial control period (1. light blue) and after DPP (2. blue) in the presence of nifedipine of the data shown in B. Whilst shift in mEPSC amplitude in the presence of nifedipine is significant (p < 0.01, Kolmogorov–Smirnov test), the shift is a decrease in mEPSC amplitude. D. As in A though in the presence of BAPTA (10 mM, to the intracellular solution). E. As in B though in the presence of BAPTA. F. Cumulative probability plot of mEPSC amplitudes for the initial control period (1. light blue) and after DPP (2. blue) in the presence of BAPTA for the data shown in E. Shift in mESPC amplitude is not significant (p = 0.937, Kolmogorov–Smirnov test). G. The fold change in mEPSC decay time constant post-DPP with respect to baseline activity (Con, n = 5; C9, n = 5; C9-Δ, n = 5). Fold changes are not significant (one-way ANOVA with Bonferroni’s multiple comparisons test). Data are consistent with the fact that average scaled mEPSC traces from pre- and post-DPP stages are superimposable. [file 13024_2021_433_MOESM11_ESM.tif]
